# Structural Modifications of Fructans in *Aloe barbadensis* Miller (Aloe Vera) Grown under Water Stress

**DOI:** 10.1371/journal.pone.0159819

**Published:** 2016-07-25

**Authors:** Carlos Salinas, Michael Handford, Markus Pauly, Paul Dupree, Liliana Cardemil

**Affiliations:** 1 Departamento de Biología, Facultad de Ciencias, Universidad de Chile, Casilla 653, Santiago, Chile; 2 Department of Plant and Microbial Biology, University of California, Berkeley, CA, 94720, United States of America; 3 Department of Biochemistry, University of Cambridge, Cambridge, CB2 1QW, United Kingdom; University of Western Sydney, AUSTRALIA

## Abstract

*Aloe barbadensis* Miller (Aloe vera) has a Crassulaceae acid metabolism which grants the plant great tolerance to water restrictions. Carbohydrates such as acemannans and fructans are among the molecules responsible for tolerating water deficit in other plant species. Nevertheless, fructans, which are prebiotic compounds, have not been described nor studied in Aloe vera, whose leaf gel is known to possess beneficial pharmaceutical, nutritional and cosmetic properties. As Aloe vera is frequently cultivated in semi-arid conditions, like those found in northern Chile, we investigated the effect of water deficit on fructan composition and structure. For this, plants were subjected to different irrigation regimes of 100%, 75%, 50% and 25% field capacity (FC). There was a significant increase in the total sugars, soluble sugars and oligo and polyfructans in plants subjected to water deficit, compared to the control condition (100% FC) in both leaf tips and bases. The amounts of fructans were also greater in the bases compared to the leaf tips in all water treatments. Fructans also increase in degree of polymerization with increasing water deficit. Glycosidic linkage analyses by GC-MS, led to the conclusion that there are structural differences between the fructans present in the leaves of control plants with respect to plants irrigated with 50% and 25% FC. Therefore, in non-stressed plants, the inulin, neo-inulin and neo-levan type of fructans predominate, while in the most stressful conditions for the plant, Aloe vera also synthesizes fructans with a more branched structure, the neofructans. To our knowledge, the synthesis and the protective role of neo-fructans under extreme water deficit has not been previously reported.

## Introduction

Plants survive extreme environments because they possess different mechanisms of protection and/or adaptation. Plants with Crassulaceae acid metabolism (CAM) are adapted to arid and semiarid environments. CAM species prevent water loss during photosynthesis by opening the stomata at night for CO_2_ fixation, when the ambient temperature drops, resulting in malic acid accumulation at night. CAM plants are also xerophytes with a thick wax cuticle covering the leaf epidermis, succulent leaves or stems capable of storing water, and extensive root systems. Among CAM species are the cacti (Cactaceae) and some Liliaceae including *Agave* spp and *Aloe* spp.

Another mechanism of adaptation to water deficit is the efficient osmotic adjustment that CAM plants perform to maximize water use efficiency [1, 2, 3, 4]. For this, CAM plants efficiently synthesize sugars, polysaccharides and other osmolytes, such as proline and glycine betaine [5, 6, 7, 4].

Among the osmolytes, water-retaining polysaccharides are responsible for the succulence features of CAM plants [8], such as acemannan found as a gel in the leaves of *Aloe* spp. In addition, fructan polysaccharides shield the plant from extreme temperatures, protecting cells against the formation of ice [9, 10, 11, 12]. They also protect against salt and lack of water, maintaining the physicochemical properties of the membranes [12, 13, 14, 15, 16, 17]. By doing so, fructans help with membrane fluidity during desiccation and extreme cold temperatures [18, 19].

Fructans are found in ~15% of angiosperms [20], as well as in fungi and bacteria [20, 21]. Fructans are fructose polymers with terminal glucose or fructose residues. Being water soluble, fructans increase the osmotic pressure inside the cell when specific enzymes hydrolyze oligo and polyfructans to smaller oligosaccharides and simple sugars [22]. Fructans are also considered storage polysaccharides in plants [15].

**Fig 1:**
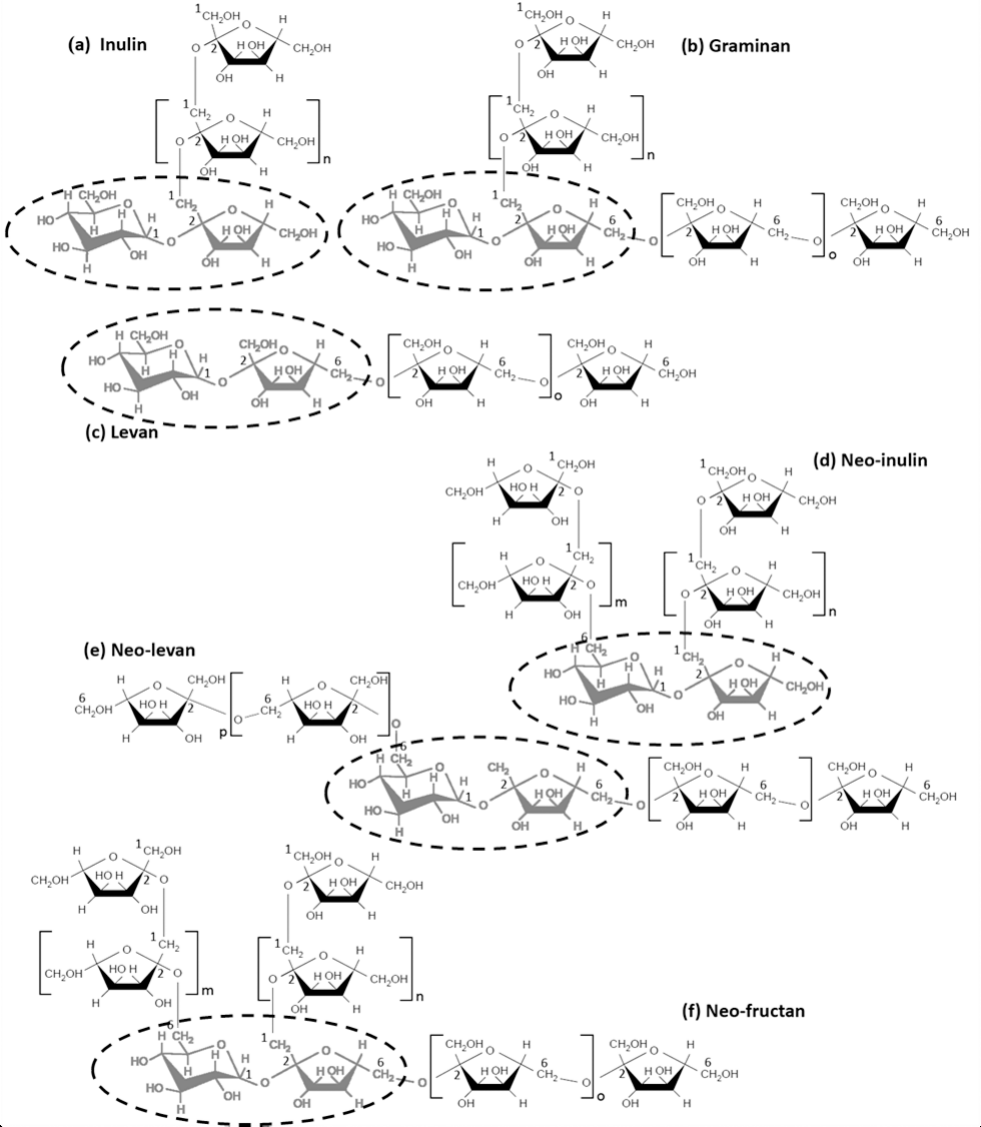
Structure of different types of fructans in plants. The sucrose molecule is encircled. Fructan molecules are named based on the position(s) of the fructan portion linked to the sucrose molecule. The fructan chains may also be further branched.

Fructans are synthesized from an initial sucrose molecule, and additional fructose residues are added to either the fructose and/or glucose moiety of the sucrose. The degree of polymerization (DP) of the fructan chain varies considerably depending on the species and environmental conditions. Chains with a DP of 3 to 10 are called oligofructans, while those with a DP of >10 are termed polyfructans. Fructans are also classified according to the type of the initial glycosidic bond of the first molecule of fructose which is bound to the sucrose. Three types of trisaccharides are distinguished: 1-kestose, 6-kestose and neo-kestose [21, 23, 24]. 1-Kestose is composed of a fructose bound to C1 of the fructose of sucrose by a β-(2→1) linkage. 6-Kestose is formed from the union of fructose to C6 of the fructose of sucrose, constituting a β-(2→6) link. In neo-kestose, the initial fructose is bound to C6 of the glucose residue of sucrose, linked by a β-(2→6) bond. In all three cases, further elongation occurs from the fructose residues, linked by β-(2→1) and/or β-(2→6) linkages. Thus, depending on the initial trisaccharide and the glycosidic bonds present, oligo and polyfructans can be classified into inulins, levans, graminans, neo-inulins, neo-levans and neo-fructans (Fig 1) [23, 25, 24].

Inulins are linear fructans, synthesized from the trisaccharide 1-kestose with additional β-(2→1)-linked fructose units [23, 24]. Commonly found in monocots, levans are sparsely-branched fructans synthesized from the 6-kestose trisaccharides, in which additional fructose residues are bound by β-(2→6) glycosidic linkages. Graminans are synthesized from 1-kestose and sucrose, producing bifurcose. This tetrasaccharide is elongated with fructose residues linked by β-(2→1) and β-(2→6) bonds, generating a branched structure. Graminans are found mainly in monocots, particularly grasses and cereals [23, 24]. Neo-inulin is found in monocots such as Asparagales, and is formed from neo-kestose which is elongated with fructose residues linked by β-(2→1) bonds. Neo-levans are also formed from neo-kestose, elongated by fructose residues with β-(2→6) linkages, and are present in Poaceae monocots [23, 24]. Finally, neo-fructans are constructed from neo-kestose, with β-(2→1) and β-(2→6) polymerized fructose molecules, generating a highly-branched structure found in monocots like *Agave* spp [23, 25].

The position and type of the glycosidic bond of the fructan chain depends on the specific activity of fructosyltransferases. To date, four types have been found in plants. Sucrose:sucrose 1-fructosyltransferase (1-SST) transfers the fructose of one molecule of sucrose to the fructose of another sucrose forming a β-(2→1) bond thereby producing 1-kestose. The elongation of 1-kestose with fructose residues is catalyzed by fructan:fructan 1-fructosyltransferase (1-FFT), synthesizing inulin. Sucrose:fructan 6-fructosyl transferase (6-SFT) generates 6-kestose by transferring a fructose residue to C6 of a fructose residue from sucrose. 6-SFT also transfers another fructose residue to the C6 of 1-kestose, forming bifurcose. Finally, fructan:fructan 6G-fructosyl transferase (6G-FFT) transfers a fructose residue to C6 of the glucose residue from sucrose, producing neo-kestose. This trisaccharide is polymerized further by 1-FFT or 6-SFT forming neo-inulins and neo-levans, respectively, and if both enzymes act, neo-fructans are produced.

Aloe vera (*Aloe barbadensis* Miller) is a CAM succulent [26] of the Asparagales order [27, 28] that evolved in the semiarid environments of North Africa. Commercially, it is used in the cosmetic, health and food industries. One of the Aloe vera products important in cosmetology and wound healing is the acetylated galactoglucomannan, acemannan, which forms a gel with wound healing and immune-modulatory properties [29]. However, to our knowledge fructans have never been reported to date in this species. Fructans are considered among the best prebiotic molecules thought to inhibit colo-rectal cancer development [30, 31].

Aloe vera was introduced in Chile at the end of the 1990´s. Although Aloe vera originates from semi-arid regions, the species has to be further adapted to the Chilean deserts, which are considered the most arid environments in the world. Because of this extreme aridity, we determined the effect of these conditions on the commercially-valuable fructans of Aloe vera. We report the presence and structures of fructans in plants subjected to severe water regimes and how fructan quantity and structure are affected under water restriction.

## Materials and Methods

### Growth conditions and induction of water stress

The tests were performed with three month old Aloe vera grown in the Experimental Station of the University of Chile during the 2006–2007 season. This site is located in the IV Region of Coquimbo, Chile (30° 17'S, 71° 15'W). Four treatments of drip irrigation, which consisted of 1600, 1200, 800 and 400 mL (the T1, T2, T3 and T4 treatments respectively) of water per plant supplied every 15 days were implemented, for three months. Thus, the only condition that was controlled in the field was the amount of water received by the plants. T1 plants were considered the control group. The volume of water for each treatment was determined by considering the field capacity (FC) of the soil, where 1600 mL corresponds to 100% FC.

### Plant material

The tips and bases of Aloe vera leaves were used. For this, the first 15 cm of the leaf sheet in contact with the stem of the plant was named as the base, whilst the 15 cm from the apical end of the blade, was designated as the tip. These leaf portions included both epidermal tissue of the leaf (photosynthetic cortex), as well as the inner pulp or gel, which is parenchyma tissue. Tip and base samples were obtained from 3 different plants (biological replicates) from each irrigation condition, from which two or three technical replicates were used for subsequent analyses. All collected samples were frozen in liquid nitrogen and stored at -80°C.

### Extraction of total sugars

Frozen leaf tips or bases (2.5 g) were macerated, then 15 mL deionized water was added and the sample boiled for 5 min. Subsequently, the extract was centrifuged (7,796 g, 15 min) at room temperature. The supernatant was collected and stored at -20°C.

### Extraction of soluble sugars

Fifteen mL of 95% ethanol (v/v) was added to 2.5 g (FW) of macerated leaf, heated (70°C, 10 min) and centrifuged (7,796 g, 15 min) at room temperature [32]. The supernatant containing the soluble sugars was collected and stored at -20°C.

### Extraction of oligofructans and polyfructans

Fructans were extracted as described [33]. Oligofructans were extracted with 10 mL ethanol 80% (v/v), using 5 g (FW) of the leaf tip or base. The leaf tissues were boiled for 5 min in the ethanol, frozen in liquid nitrogen and macerated. The macerated sample was mixed again with the ethanol-treated leaf from the first extraction and boiled again. The sample was centrifuged (1,949 g, 5 min) at room temperature. The supernatants were pooled, named as the oligofructan extract, and stored at -20°C. To extract polyfructans, the pellet obtained from the last centrifugation of the oligofructan extraction was re-suspended in 10 mL deionized water and heated (15 min, 60°C). The extract was centrifuged (1,949 g, 5 min) at room temperature. The supernatant was collected and a second extraction of the pellet obtained from the first centrifugation was performed by resuspending in 3 mL deionized water and boiling. Both supernatants were pooled, designated as the polyfructan extract and stored at -20°C.

### Quantification of total and soluble sugars

Total and soluble sugars were quantified by the anthrone reagent [34]. The quantifications were undertaken in test tubes using 10 μL of each sample plus 990 μL deionized water, mixing and supplementing with 2 mL anthrone solution. The samples were incubated for 10 min on ice and then at room temperature for another 10 min, before being measured in a spectrophotometer at 620 nm (Thermo Spectronic Genesys 20, model 4001/4). A standard curve was performed using 1 mg/mL glucose, to determine glucose concentrations, which were expressed as mg/g DW.

### Quantification of oligofructans and polyfructans

Oligofructans and polyfructans were quantified using an anthrone method, optimized for greater sensitivity for ketoses [35, 36]. The anthrone reagent was prepared from 300 mg anthrone dissolved in 36 mL deionized water and 114 mL concentrated H_2_SO_4_. For quantification, 10 or 20 μL of each sample and 240 μL deionized water were mixed with 2.5 mL anthrone reagent. The solutions were shaken vigorously and incubated (37°C, 45 min). After 10 min at room temperature, absorbance was read at 618 nm. The results obtained were compared with the standard curve (0.2 mg/mL fructose) to determine fructose concentrations and expressed as mg/g DW.

### Fructan enrichment

Ionic exchange chromatography was used for fructan enrichment without pigments [37]. The extracts were previously-neutralized using NH_4_OH. Neutralized samples were passed at room temperature, through a column with 6 mL cationic resin (Dowex 50x8-200, Sigma-Aldrich) and then through a column with 6 mL anionic resin (Dowex 1x8-200, Sigma-Aldrich), using deionized water as the mobile phase (1.6 mL/min). The fructan concentration of each fraction (40 μL), was quantified by the anthrone assay. Those fractions containing fructans were concentrated on a rotary-evaporator, (Buchi, model RE 111) and freeze-dried and stored at room temperature.

### Protein determination

For the extraction and determination of proteins, 5 g of leaf tissue were frozen in liquid N_2_ and ground in a coffee grinder. The fine powder was transferred into a tube with 10 mL of extraction buffer (100 mM monobasic potassium phosphate, pH 7.0; 2 mM EDTA, and 1% of polyvinylpyrrolidone-40 (PVP-40)). The tube was vigorously shaken for 30 s. Once the tissue was homogenized, the mix was centrifuged at 8.645 g for 12 min at 4°C. The supernatant was collected and stored at −20°C until use. Total proteins were quantified by the Bradford method [38].

### Dry weight determination

The dry weight was determined by taking 5 g of fresh leaves from which soluble sugars, total sugars and fructans were extracted. Leaves were dried in an oven at 50°C for two days, and then weighed. All sugars, fructans and protein quantities are specified in the Results as mg of the respective carbohydrate per g of dry weight.

### Thin Layer Chromatography (TLC) of fructans

The oligofructans were separated by TLC to determine their DP, according to Spollen and Nelson [39]. Aliquots (12 μg) of the purified fructans were applied to a TLC plate of silica gel 60 F254 20x20 cm (Merck). On the same plates, standards were loaded: fructose (40 μg, Merck), sucrose (40 μg, Merck), kestose trisaccharide (20 μg, Megazyme), tetrasaccharide (20 μg, Megazyme) and pentasaccharide (20 μg, Megazyme). The mobile phase consisted of 1-butanol:acetic acid:water (55:30:15), in which the plate was run 3 times. Staining and development of the plate were performed by spraying a solution of urea-phosphoric acid [40], heating (150°C, 10 min) and photographing in white and UV light (366 nm).

Quantitative chromatographic analysis of TLC plate extracts of oligo- and polyfructans was carried out with MCID Analysis software version 7.0, in order to estimate the amount of fructan in the samples without interference from sucrose and fructose.

### Determination of fructan length by MALDI-ToF-MS in plants under water restrictions

Enriched fructans oligosaccharides were analysed by MALDI-ToF-MS using a 4700 Proteomics Analyser (Applied Biosystems), as described by Maslen et al. [41]. The matrix was 2,5-dihydroxybenzoic acid (10 mg ml^−1^ dissolved in 50% MeOH). Three different T1 plants and three different T4 plants were used for this analysis. The MALDI-ToF-MS was done with three technical replicates. The results were statistically analyzed by Student’s t test.

### Analysis of fructans by gas chromatography coupled with mass spectrometry (GC-MS)

To determine the monosaccharide residue composition of the fructan, as well as to quantify the concentrations of fructans in leaves, acetylated alditol derivatization was performed [42, 43]. This method hydrolyzes the polysaccharides to monosaccharides, which are then reduced with sodium borohydride (NaBH_4_). The alditols were per-O-acetylated and extracted in ethyl acetate and 4 mL water. Fifty μL were taken for analysis by GC-MS (Agilent 7890A GC System with an automated sample injection, Agilent 7683 Automatic Liquid Sampler and Agilent 5975 MS). The column employed was a Supelco SP-2380 (Sigma-Aldrich, 30 m x 0.25 mm x 0.20 μm). GC / MSD ChemStation software version E.02.00.493 from Agilent Technologies was used to analyze the chromatograms.

### Analysis of the glycosidic linkages present in fructans

To determine the types of glycosidic bonds present in the fructans, partially methylated alditol acetates (PMAA) were generated from the sugar residues obtained after hydrolysis of the polysaccharides and analyzed by GC-MS [42, 44] with certain modifications [45]. For methylation, 150 μL fructan solution in DMSO (1 mg/mL) was lyophilized, 200 μL DMSO added, and stirred at room temperature for 15 min. Subsequently, 200 μL of 50% NaOH solution in DMSO and 100 μL methyl iodide was added according to [45]. Next, nitrogen was introduced and the solution vortexed, sonicated for 5 min and stirred for 30 min. To stop the methylation reaction, 2 mL deionized water were added and gently stirred. Finally, nitrogen was bubbled through the solution until the cloudy solution became clear.

### Reduction to alditols

For the reduction to per-methylated alditols, the protocol used was similar to that employed for monosaccharide compositional analysis except that NaBD_4_ was used.

### Extraction of per-O-methylated acetylated alditols

The per-O-methylated acetylated alditols were extracted in 100 μL ethyl acetate. The samples were subsequently diluted with 50 μL acetone and placed in closed GC-MS vials.

### Determination of glycosidic linkages of fructans by GC-MS

The molar percent (mol %) of each type of glycosidic linkage present in the samples was calculated by integrating each peak area obtained in the fructan chromatograms. The samples were injected into a GC coupled to a quadrupole MS. The GC-MS was equipped with a Supelco SP-2380 (30 m x 0.25 mm x 0.25 μm) column. The He flow rate was 1.5 mL/min. The following temperature program was used: initial hold (160°C, 2 min), a 20°C/min ramp to 200°C and hold (5 min), a 20°C/min ramp to 245°C and hold (12 min), spike to 270°C and hold (5 min) before cooling to the initial temperature [46]. Due to tautomerization, the peaks corresponding to mannose and glucose were defined as corresponding to fructose and glucose of fructans, based on both the fragments given and by the retention times of standards. The fructan glycosidic linkages were compared with those reported by Mancilla-Margally and López [25] for those of *Agave tequilana*. Data are the average of three independent fructan preparations.

### Statistical analyses

Analyses were carried out by using one way ANOVA to test for differences between water treatments (T1–T4). Significant differences were further tested with Tukey’s multiple range test. The differences were considered significant at P ≤ 0.05. The differences between leaf regions (tips and bases) were evaluated by the Student t test, where *: P < 0.05; **: P < 0.005; ***: P < 0.001. All analyses were performed with GraphPad Prism 5.

## Results

### Total and soluble sugar quantification

**Fig 2:**
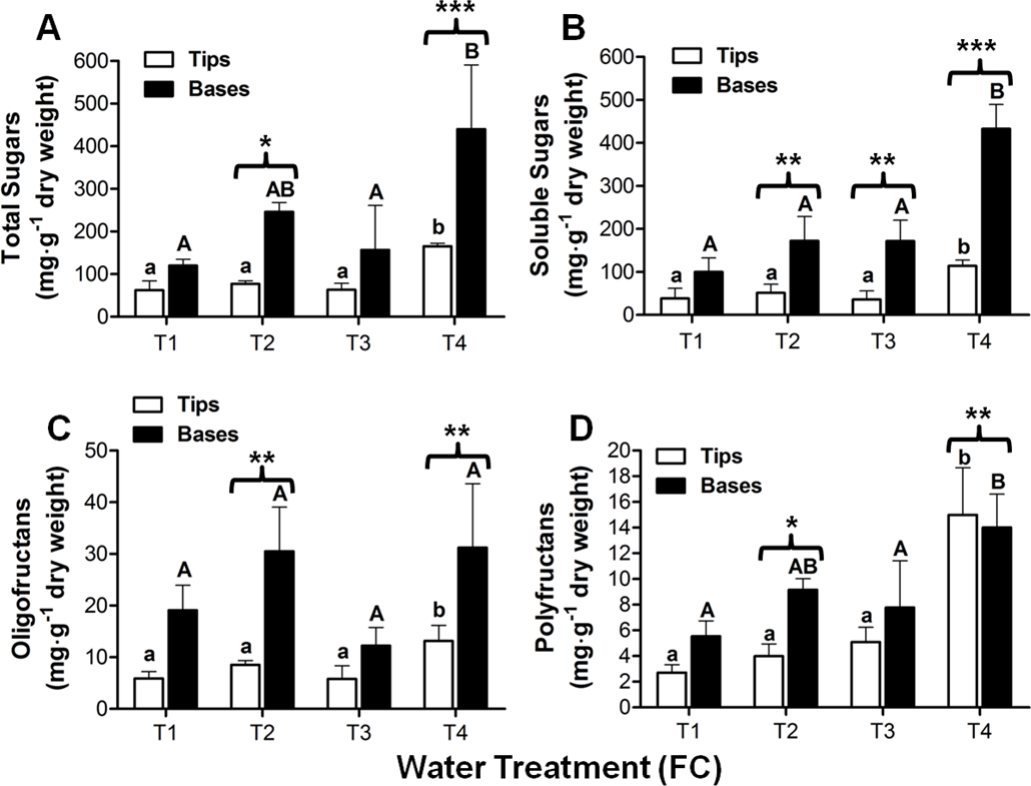
Total, soluble sugars, oligofructans and polyfructans in leaf tips and bases of Aloe vera plants. Plants were subjected to four different water treatments as described. T1 plants corresponding to 100% FC were the control group. **(A)** Total sugars. **(B)** Soluble sugars. **(C)** Oligofructans and **(D)** Polyfructans. Each column represents the average of three independent biological samples (three different plants) and two technical replicas with their respective standard deviation. The asterisks indicate a significant difference between leaf tips and bases. Student t test (*: P < 0.05; **: P < 0.005; ***: P < 0.001). Different letters denote significant differences between treatments (Tukey´s test, P ≤ 0.05). Capital letters denote differences between leaf bases and lower case letters denote differences between leaf tips.

The concentrations of total and soluble sugars in the leaf tip and base of Aloe vera plants subjected to 4 water treatments (T1: 1600, T2: 1400, T3: 800 and T4: 400 mL/plant every 15 days for 3 months) was determined by the anthrone test. Total sugars increase in both leaf tips and bases upon decreased water treatment. At the base the amount of total sugars in plants in low water, T4 treatments, is almost 5.5 times the amount in high water T1 treated plants (Fig 2A).

Since soluble sugars are osmolytes that can be associated with protection against water stress, these sugars were also analyzed in plants subjected to water deficit (Fig 2B). Most of the total sugars in the samples are represented by soluble sugars (80–90%). The soluble sugars increase more in bases than in tips upon reducing the watering regime, rising 3.7-fold in T4 plants compared to the control plants (T1).

### Fructan quantification

Fructans are water soluble and are considered osmolytes. Specific extractions of oligofructans and polyfructans were undertaken. Both types of fructans increase in the leaf tip and base with water stress. In bases, oligofructans increase 52% in T2 and T4 treatments compared with T1, while in tips the increment is 60% in T4 compared with T1 (Fig 2C). Polyfructans also increase with water deficit. Unlike the oligofructans, polyfructans increase in tips more than in bases. In tips, the polyfructans increase in T4 treated plants 6.2 times the basal amount in T1 plants, while in bases, the amount in T4 plants is 2.33 times the amount in T1 plants (Fig 2D).

### Total protein quantification

**Fig 3:**
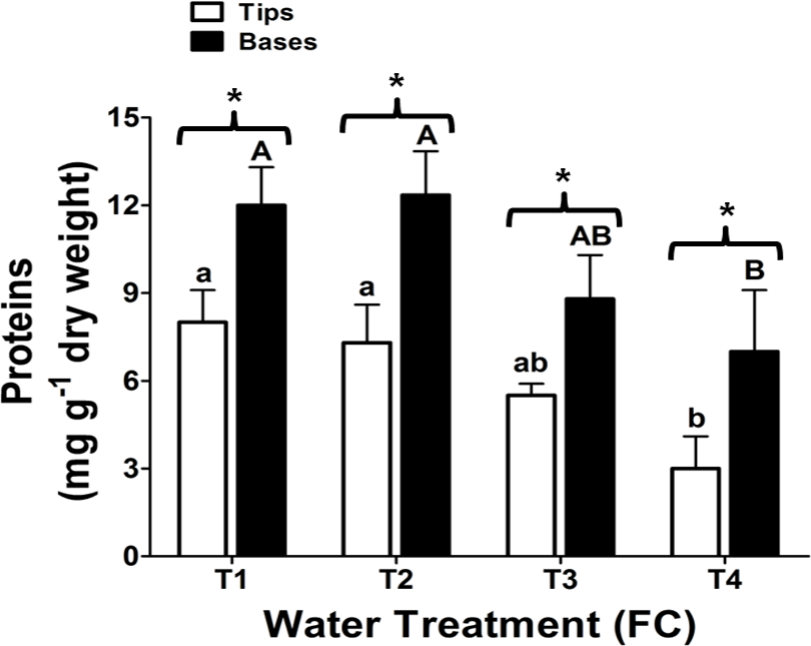
Total proteins in leaf tips and bases of Aloe vera plants. Plants were subjected to four different water treatments as described previously. Each column represents the average of three independent biological samples (three different plants) and two technical replicas with their respective standard deviation. The asterisks indicate a significant difference between leaf tips and bases. Student t test (*: P < 0.05). Different letters denote significant differences between treatments (Tukey´s test, P ≤ 0.05). Capital letters denote significant differences between leaf bases and lower case letters denote differences between leaf tips.

In order to determine whether the effect of water stress on Aloe vera affected macromolecules other than fructans, protein levels were quantified in leaves subjected to drought conditions. Fig 3 shows that the total protein concentration decreases as the water deficit increases in leaves of Aloe vera plants. In the tips, the total amount of proteins decreased more from T1 to T4 than in the bases of the leaves. In T4 plants, the tips contain 62.5% less proteins than T1 plants, while the bases contain only 41.7% less proteins than the control plants.

### Qualitative analysis of oligo and polyfructans by TLC

#### Oligofructans

**Fig 4:**
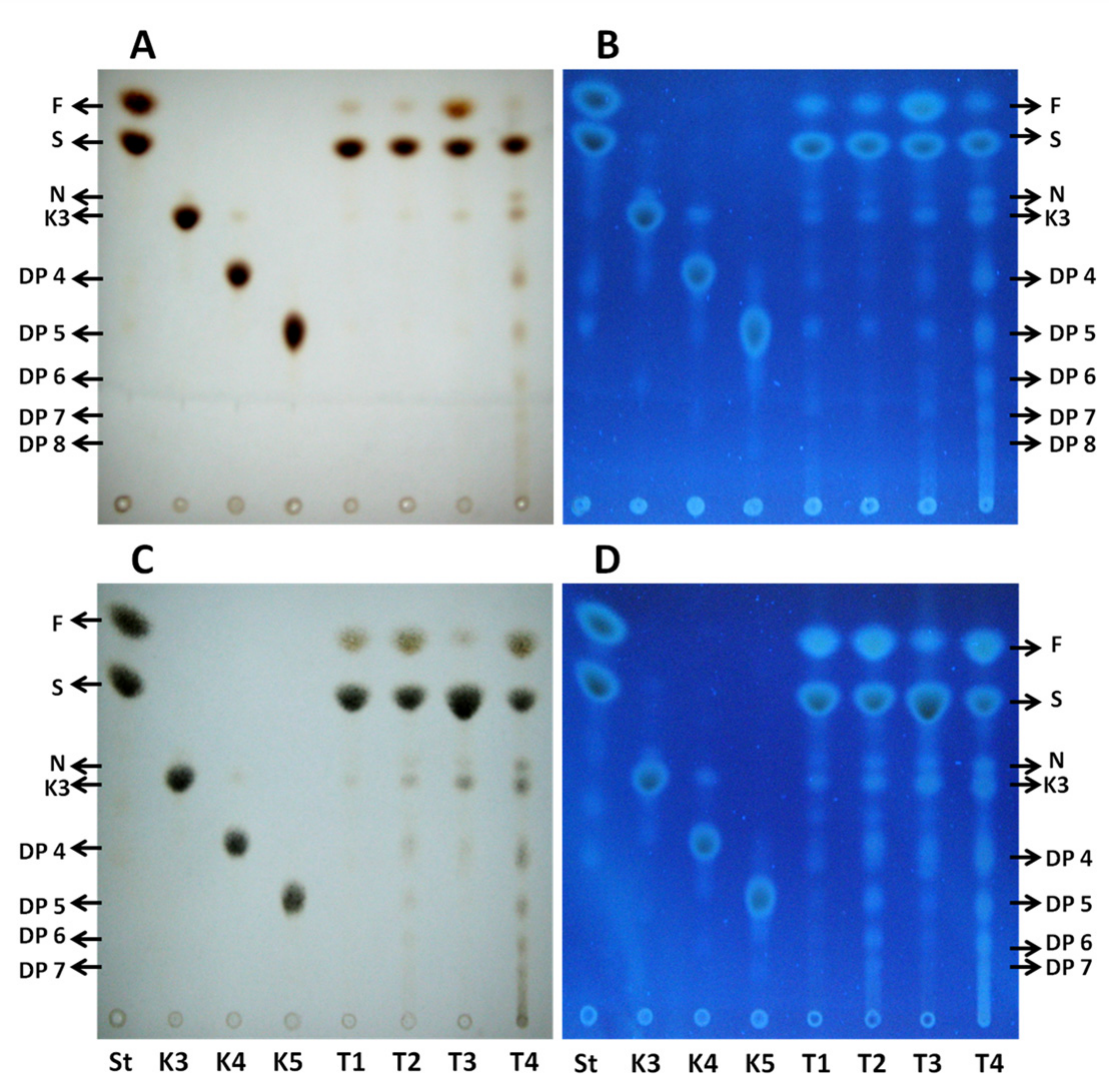
TLC analyses of oligofructans extracted from leaf tips and bases of Aloe vera plants. Oligofructans from leaf tips **(A** and **B)** and bases **(C** and **D)** were extracted from plants subjected to the four water treatments. **A** and **C**, oligofructans stained with urea-phosphoric acid observed in the silica gel plate under white light. **B** and **D,** oligofructans seen in the silica gel plate under UV light (366 nm). S: standards of fructose (F) and sucrose (S). K3, K4, K5: inulin oligosaccharide standards. K3: 1-kestose (trisaccharide), K4: tetrasaccharide, K5: pentasaccharide. N: neo-kestose, trisaccharide of the neo-fructan series.

**Fig 5:**
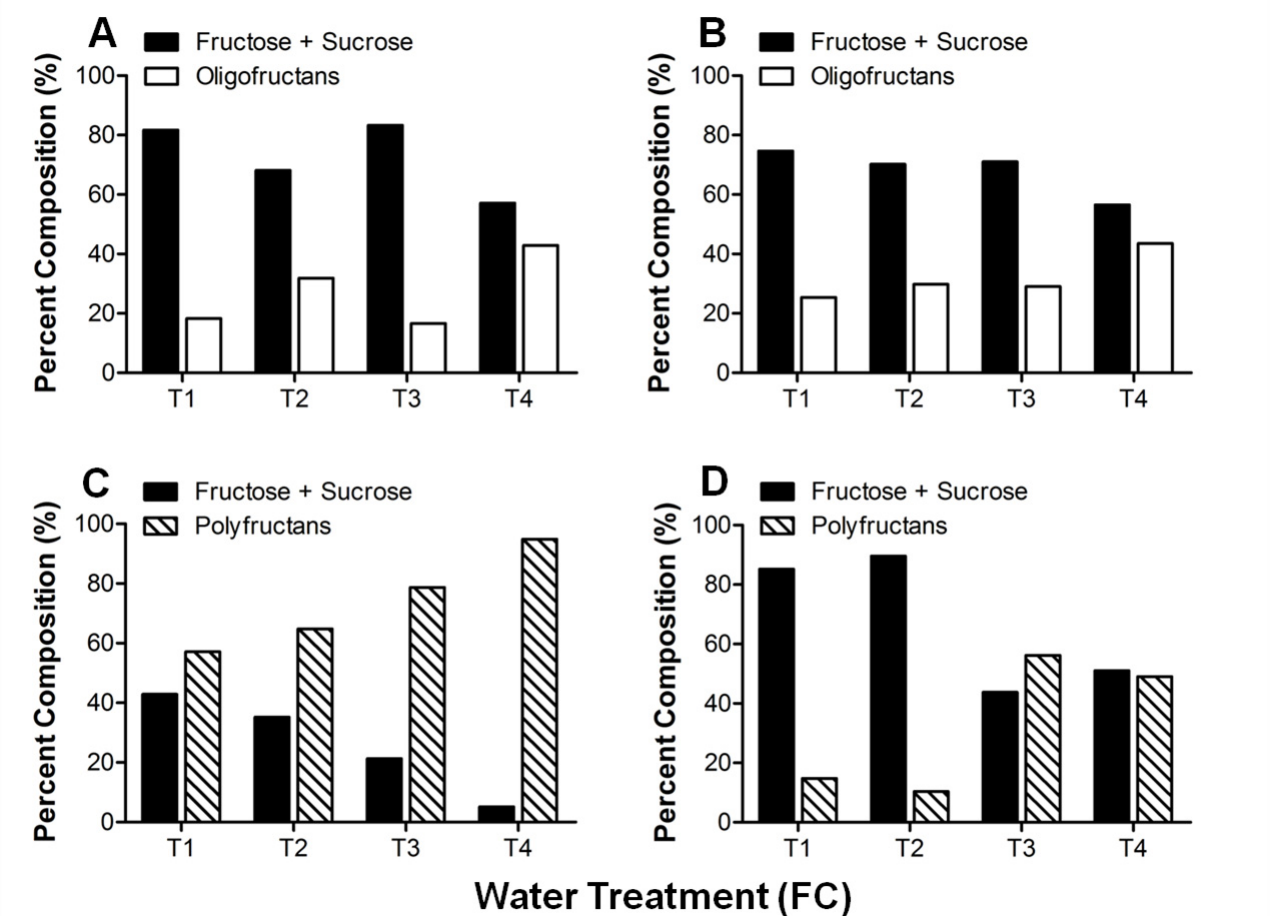
Sugar composition of Aloe vera fructan extracts by TLC analysis. Fructan extracts from Aloe vera leaf tips (A and C) and bases (B and D) were loaded onto a TLC plate and analyzed to determine the sugar composition of each sample. The percent composition of the different fructan samples was determined by calculating the surface area and intensity of the sugar spots obtained on a TLC plate. Oligofructans from leaf tips (A) and bases (B) and polyfructans from leaf tips (C) and bases (D) were extracted from different plants subjected to the four water treatments. This analysis was performed on a single TLC plate for leaf tips and for bases. Spot area was measured three times, with no differences observed between readings.

Fig 4 shows the TLC analysis of the oligofructans present in tips and bases of leaves of Aloe vera plants subjected to the water treatments. Visualizing the plates under UV facilitated the identification of several sugars (1- kestose, K; neo-kestose, N; Fig 4B and 4D). In tip tissue, the sucrose spots are very intense, while the signal corresponding to fructose is most intense in the T3 treatment (Fig 4A and 4B). In leaf bases, the fructose spots are similar in T1, T2, and T4 plants and less intense in T3 plants, seemingly replaced by sucrose in the latter treatment (Fig 4C and 4D). In tips and bases, kestose and neo-kestose were detected in increasing amounts from T1 to T4. Therefore, in general, oligofructans increase significantly, and appear to have a higher DP in tips and bases of plants subjected to greater water deficit (T3 and T4). From the TLC analysis, the percent of fructose + sucrose and the percent of oligofructans were estimated for leaf tips and bases as explained in Materials and Methods (Fig 5A and 5B). With increased water deficit, the percent of fructose+sucrose decreases while that of oligofructans increases in tips and bases, probably because fructose and sucrose are used for the synthesis of these polymers.

#### Polyfructans

**Fig 6:**
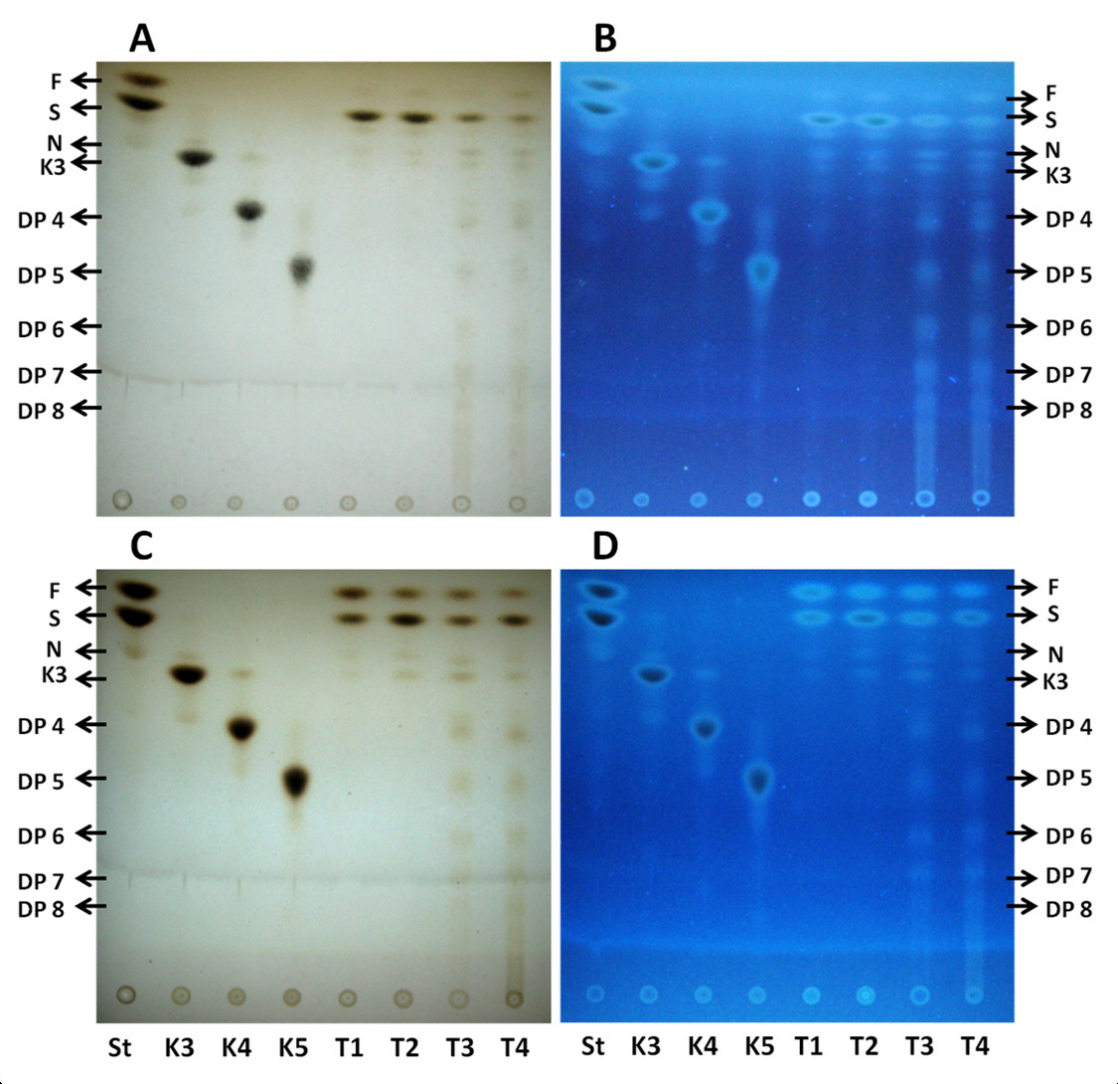
TLC analysis of polyfructans extracted from leaf tips and bases of Aloe vera plants. Polyfructans from leaf tips **(A** and **B)** and bases **(C** and **D)** were extracted from plants subjected to the four water treatments. **A** and **C**, polyfructans stained with urea-phosphoric acid seen in the silica gel under white light. **B** and **D,** polyfructans seen in the silica gel plate under UV light (366 nm). S: standards of fructose (F) and sucrose (S). K3, K4, K5: inulin oligosaccharide standards. K3: 1-kestose (trisaccharide), K4: tetrasaccharide, K5: pentasaccharide. N: neo-kestose, trisaccharide of the neo-fructan series.

In leaf tips and bases (Fig 6A, 6B, 6C and 6D), there is a greater number of spots with sugars of a higher DP in T3 and T4 treatments, than in T1 and T2 treatments. The presence of sucrose is observed in all treatments, particularly in T1 and T2 plants. In tips (Fig 6A and 6B), the fructose spots are of low intensity under all treatments, and more intense in bases (Fig 6C and 6D). 1-Kestose and neokestose are detected in all treatments, with the neokestose more prevalent in tips and bases of T3 and T4 plants.

The percent of polyfructans in tips is estimated to be higher than that in bases under all water treatments (Fig 5C and 5D). In tips and bases, the greatest percent of polyfructans are found in leaves subjected to severe water deficit. In tips (Fig 5C), the percent of polyfructans increases by 56% and 95% in the T3 and T4 plants, respectively. In the bases, the polyfructans rise by 58% and 55% in T3 and T4 plants, respectively (Fig 5D). As for oligofructans, the percent of fructose+sucrose decreases with water deficit, in both parts of the leaves. Specifically, fructose+sucrose falls by 88% in the tips and 60% in the bases, in T4 treated plants compared to T1 samples (Fig 5C and 5D).

### DP determination of fructans by MALDI-ToF-MS

**Fig 7:**
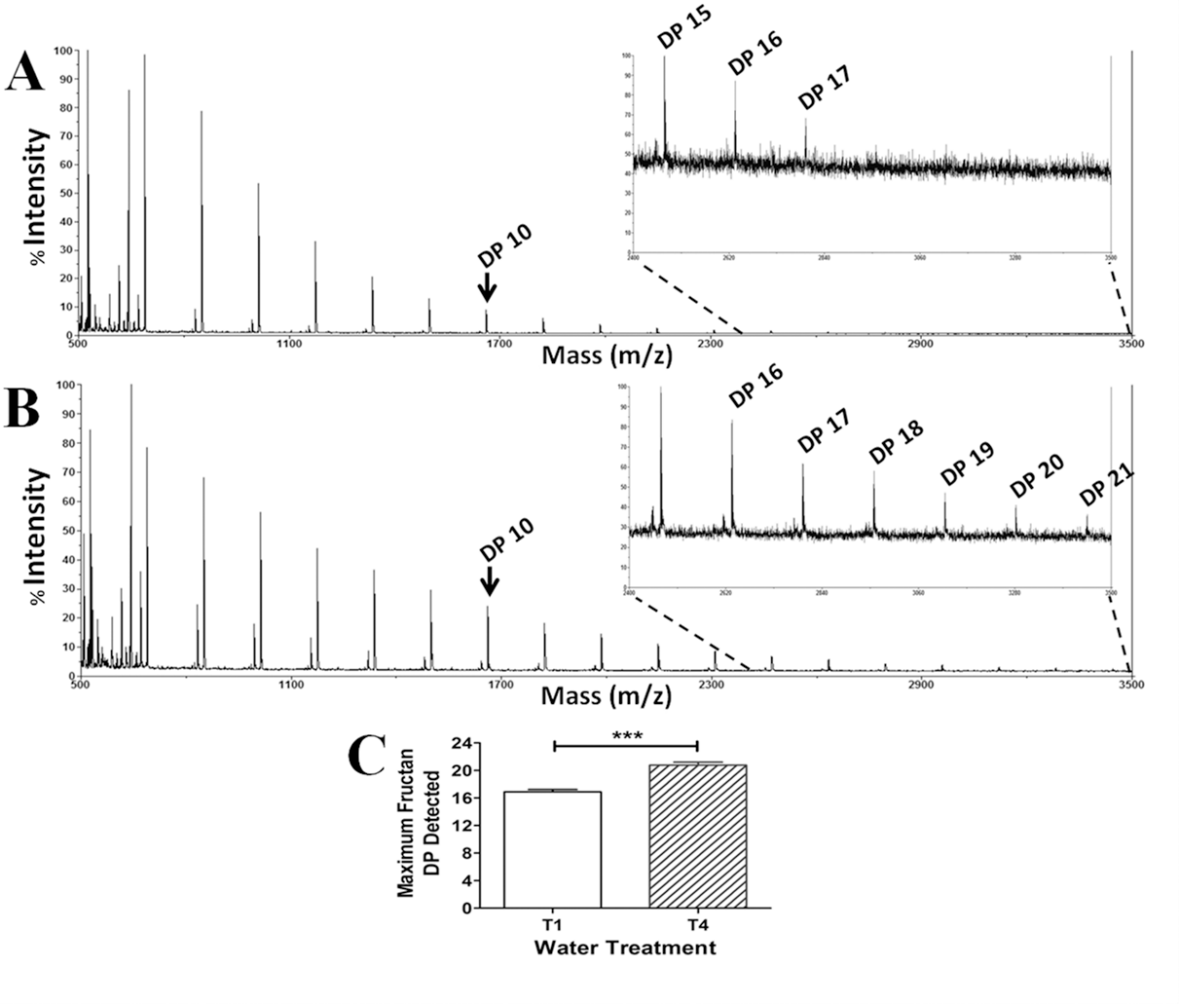
Determination by MALDI-ToF-MS of Fructan DP from Aloe vera plants subjected to different irrigation treatments. The spectra from T1 **(A)** and T4 **(B)** plants show a clear increase of DP from fructans of Aloe vera plants grown in T4 compared to T1 treatment. An insert of a sub-section (between 2400 and 3500 m/z) of each chromatogram is shown by the dotted lines. An arrow indicates a fructan of DP 10 present in both chromatograms **(A)** and **(B)**. **(C)** shows a significant increase in the average fructan DP from T1 plants to T4 plants (Student t-test, ***: P < 0.001).

To corroborate the finding by TLC that the DP of fructans from T4 has higher length, samples from T1 and T4 plants were analyzed by MALDI-ToF-MS. It was found that water restrictions increased the maximum DP of fructans from 17 in T1 plants to 21 in T4 plants, data that was significantly different between these two groups of plants (Fig 7A, 7B and 7C).

### Sugar composition analyses of oligo and polyfructans by GC-MS

#### Oligofructans

**Fig 8:**
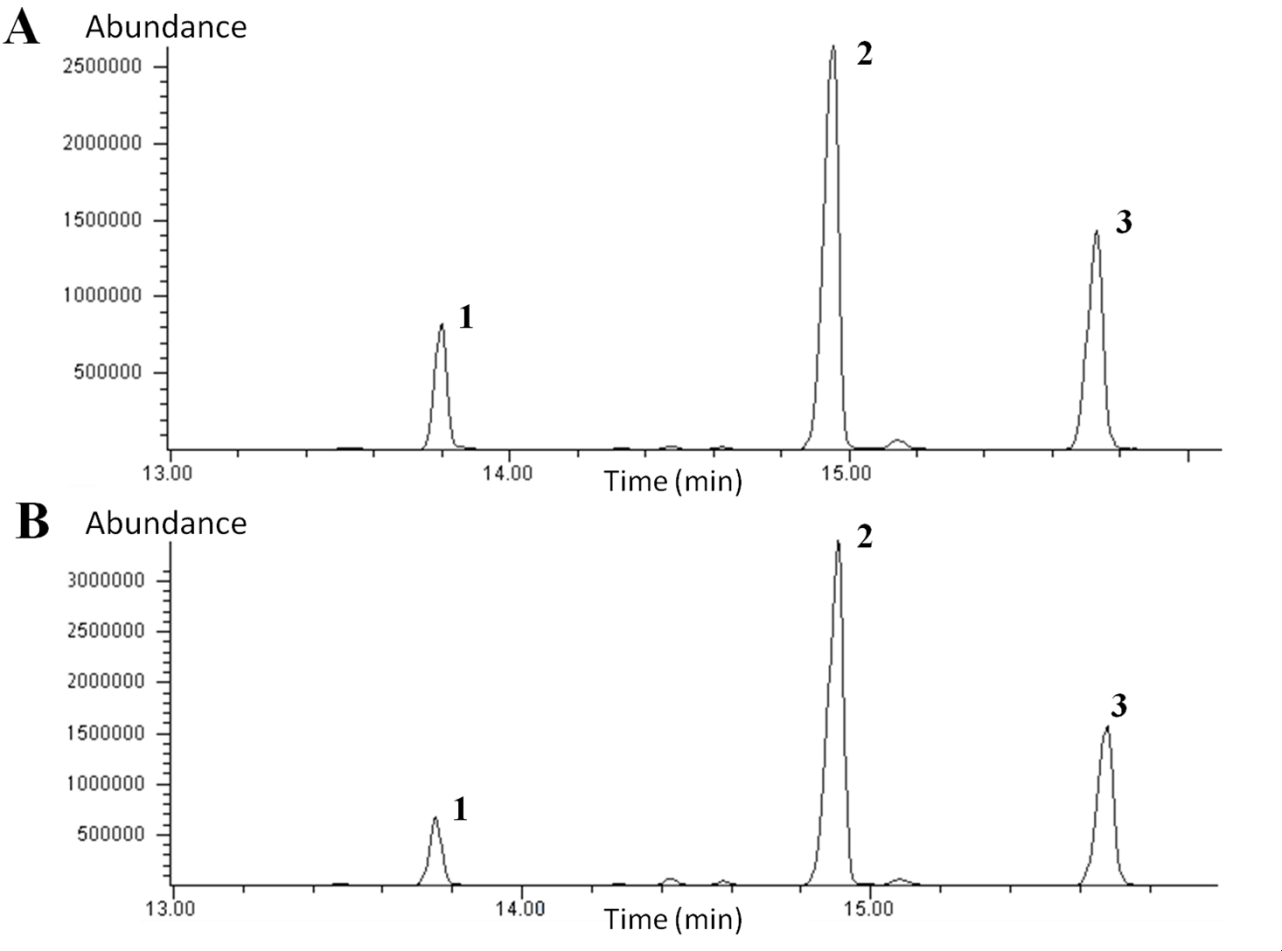
Gas chromatograms of the sugar components of oligofructans from Aloe vera leaf tips and bases. The sugar components of oligofructans, glucose and fructose were derivatized to alditol acetates. **A)** Chromatogram of the alditol acetates from the leaf tip of Aloe vera plants. **B)** Chromatogram of the alditol acetates from the leaf base of Aloe vera plants. Peak 1: mannose. Peak 2: glucose. Peak 3: inositol, the internal standard.

The sugar components of the oligofructans in tips (Fig 8A) and bases (Fig 8B) were identified based on their alditol acetate derivative quantification by GC-MS. Fructose tautomerisation gives rise to two per-O-acetylated peaks, mannose and glucose, which were the only peaks detected in the chromatograms. The peak area of glucose was 3.66 times larger than the mannose peak in tips and 4.48 times larger in bases. The mass spectra of these peaks produced the following ionized fragments: 73, 103, 115 (the fragment of greatest intensity), 128, 139, 145, 187, 217, 259, 289 and 361, which are the characteristic fragments of the alditol acetate derivatives of both hexopyranoses (mannitol and sorbitol, in the case of mannose and glucose, respectively).

From the chromatogram results, oligofructans from the purified samples obtained from leaves of plants subjected to the four water treatments were quantified as the sum of mannose and glucose (Table 1). At a higher water deficit, the quantity of oligofructans increases in tips and bases. For example, oligofructans are 2.4-fold and 1.8-fold more abundant in T4 samples compared to T1 samples, in tips and bases, respectively.

**Table 1:** Quantification of oligofructans purified from leaves of Aloe vera plants subjected to different water treatments.

Sample	Mannose (mg/g DW)	Glucose (mg/g DW)	Total Oligofructans (mg/g DW)
**TT1**	1.55 ± 0.05 a	5.60 ± 0.29 a	7.15 ± 0.34 a
**TT2**	1.78 ± 0.07 b	6.56 ± 0.26 b	8.34 ± 0.32 b
**TT3**	0.69 ± 0.03 c	6.78 ± 0.47 b	7.47 ± 0.50 ab
**TT4**	4.20 ± 0.10 d	12.76 ± 0.30 c	16.96 ± 0.40 c
**BT1**	3.86 ± 0.11 a	25.48 ± 1.00 a	29.34 ± 2.97 a
**BT2**	6.33 ± 0.08 b	39.71 ± 1.18 b	46.04 ± 4.45 b
**BT3**	4.98 ± 0.10 c	20.31 ± 0.41 c	25.29 ± 3.21 a
**BT4**	8.92 ± 0.05 d	41.87 ± 0.91 b	50.79 ± 5.72 b

The amounts of mannose, glucose and total oligofructans (sum of mannose and glucose) are shown (n = 3; mean ± SD). TT1-TT4: leaf tip samples with their respective water treatment. BT1-BT4: leaf base samples with their respective water treatment. Different lower case letters denote significant differences between treatments (Tukey´s test, P ≤ 0.05).

#### Polyfructans

The enriched polyfructans show a proportion of mannose to glucose for the tip and base similar to those of oligofructans upon all water treatments (chromatograms not shown, Table 2). In tips, there was a difference of 3.4 times between mannose and glucose, while in bases the difference was 5.0 times. Polyfructans, like oligofructans, significantly increase with water deficit particularly in T4 plants. In T4 tips, 4.22-fold more polyfructans were found compared to T1 plants, whereas in the base this difference was 2.36-fold.

**Table 2:** Quantification of polyfructans purified from leaves of Aloe vera plants subjected to different water treatments.

Sample	Mannose (mg/ g DW)	Glucose (mg/ g DW)	Total Polyfructans (mg/g DW)
**TT1**	0.79 ± 0.02 a	3.01 ± 0.08 a	3.80 ± 0.10 a
**TT2**	1.25 ± 0.09 b	4.84 ± 0.47 b	6.10 ± 0.56 b
**TT3**	1.55 ± 0.10 b	4.43 ± 0.16 b	5.98 ± 0.24 b
**TT4**	4.71 ± 0.23 c	11.38 ± 0.35 c	16.09 ± 0.57 c
**BT1**	1.74 ± 0.07 a	9.18 ± 0.32 a	10.92 ± 0.38 a
**BT2**	1.21 ± 0.01 b	7.67 ± 0.12 b	8.87 ± 0.14 b
**BT3**	3.74 ± 0.22 c	11.06 ± 0.61 c	14.80 ± 0.83 c
**BT4**	4.75 ± 0.10 d	20.97 ± 0.33 d	25.72 ± 0.42 d

The amounts of mannose, glucose and total polyfructans (sum of mannose and glucose) are shown (n = 3; mean ± SD). TT1-TT4: leaf tip samples with their respective water treatment. BT1-BT4: leaf base samples with their respective water treatment. Different lower case letters denote significant differences between treatments (Tukey´s test, P ≤ 0.05).

### Determination of the glycosidic bonds of fructans by GC-MS analyses of their partial methylated alditol acetates (PMAA)

The glycosidic linkages present in the fructans of plants subjected to the different water treatments were identified and quantified by GC-MS of their PMAA derivatives. For this, the oligo- and polyfructans were partially methylated, followed by the release of methyl sugars after hydrolysis of the methylated polymers, and derivatization to alditol acetates, as described in the methodology. All the chromatograms showed a maximum of seven peaks which were identified from the Rt and mass spectrometric analyses (Table 3).

**Table 3:** Identification of the PMAA sugar residues of fructans from the leaf tips and bases of plants of Aloe vera.

Peak	Rt	Derivatized Sugar Residue	Linkage Types
**1**	11.73	2,5-di-O-acetyl-(2-deuterium)-1,3,4,6-tetra-O-metyl-D-mannitol	terminal fructose
**2**	11.97	2,5-di-O-acetyl-(2-deuterium)-1,3,4,6-tetra-O-metyl-D-sorbitol	terminal fructose
**3**	12.90	1,5-di-O-acetyl-(1-deuterium)-2,3,4,6-tetra-O-metyl-D-sorbitol	terminal glucose
**4**	15.60	1,2,5-tri-O-acetyl-(2-deuterium)-3,4,6-tri-O-metyl-D-mannitol and 2,5,6-tri-O-acetyl-(2-deuterium)-1,3,4-tri-O-metyl-D-hexitol	1-fructose and 6-fructose
**5**	15.72	1,2,5-tri-O-acetyl-(2-deuterium)-3,4,6-tri-O-metyl-D-sorbitol	1-fructose
**6**	16.60	1,5,6-tri-O-acetyl-(1-deuterium)-2,3,4-tri-O-metyl-D-sorbitol	6-glucose
**7**	20.22	1,2,5,6-tetra-O-acetyl-(2-deuterium)-3,4-di-O-metyl-D-hexitol	1,6-branched fructose

The correlative numbers (Peak) correspond to the elution order of the sugar derivatives from the GC column. Rt: retention time (minutes).

#### Glycosidic linkages of oligofructans

**Fig 9:**
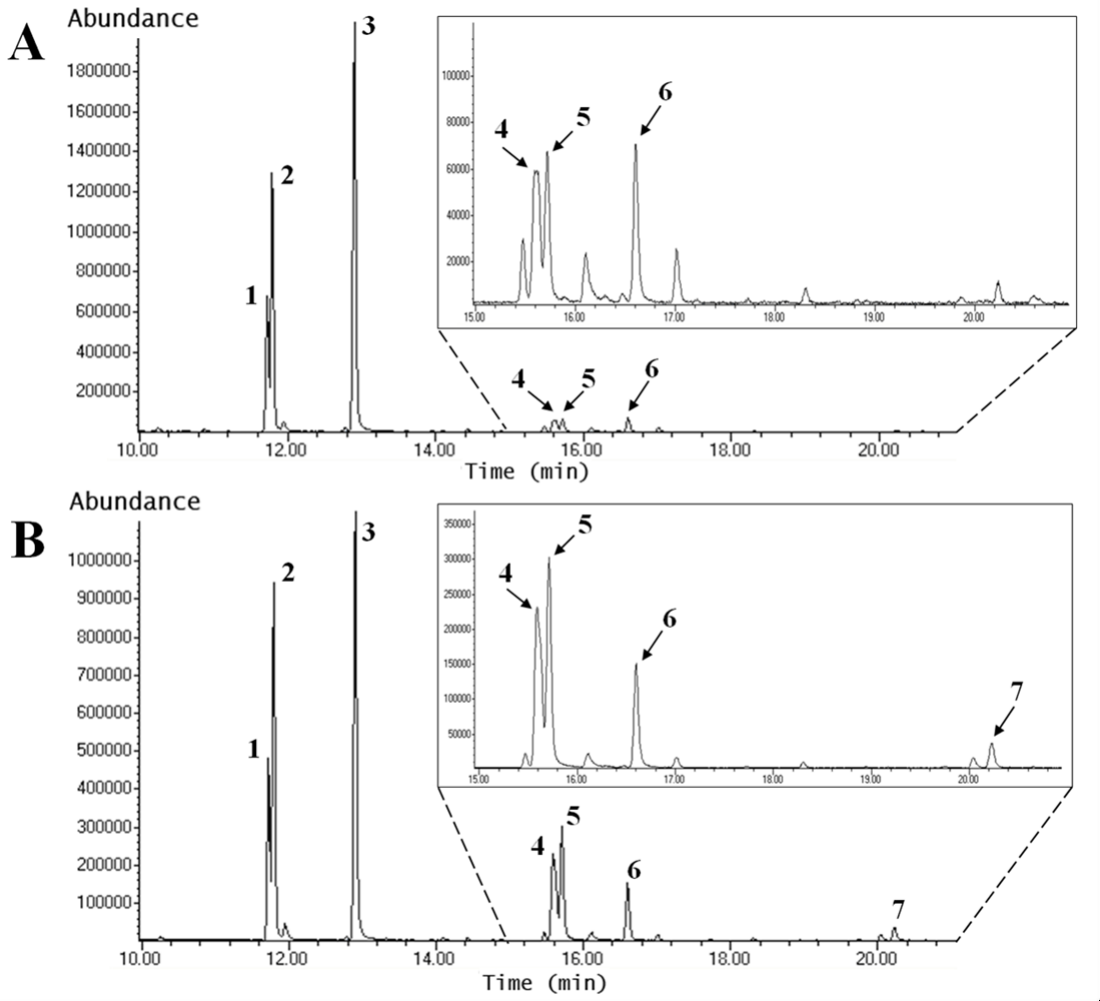
PMAA profiles of oligofructans from Aloe vera leaf tips of plants under different irrigation conditions. The figure shows the results for T1 **(A)** and T4 **(B)** plants. **(A)** Chromatogram of PMAA oligofructans from leaf tips of Aloe vera T1 plants. **(B)** Chromatogram of PMAA oligofructans form leaf tips of Aloe vera T4 plants. Peaks: 1 and 2: terminal fructose, 3: terminal glucose, 4: 1-fructose and 6-fructose, 5: 1-fructose, 6: 6-glucose and 7: 1,6-fructose. The insert corresponds to the amplified chromatogram region shown with dotted lines.

The chromatograms of PMAA derivatives from oligofructans in tips are shown for leaf tips (Fig 9A, T1 tips; Fig 9B, T4 tips). Chromatograms corresponding to T1 plants showed six peaks (Fig 9A), while there are seven peaks in the chromatograms corresponding to T4 plants (Fig 9B).

From the retention times and mass spectrometric analyses, the glycosidic linkages of the oligofructan peaks were identified (Table 4). The glycosidic linkages found were interpreted based on research reported for fructans by Carpita and Shea [47] and Mancilla-Margalli and López [25]. Terminal fructose is present in the fructan chain, whilst terminal glucose corresponds to the glucopyranose present in the initial sucrose linked at C1 to a fructofuranose. 1-Fructose is linked by β-(2→1) bonds to other fructose residues, whereas 6-fructose is a fructose residue linked by β-(2→6) bonds to another fructose. 6-Glucose is a glucose linked at its C6 to C2 of a fructose residue and is characteristic of the neo-fructan series. Finally, 1,6 fructose gives rise to branched fructans as the fructose is linked to other fructoses by β-(2→1) and β-(2→6) bonds. Note that 1,6 fructose was found only in tips and bases of T4 plants.

**Table 4:** Glycosidic linkage composition (molar %) of the oligofructans of leaf tips and bases of Aloe vera plants subjected to different water treatments.

	Water Treatment (molar %)							
Glycosidic linkages	OTT1	OTT2	OTT3	OTT4	OBT1	OBT2	OBT3	OBT4
**Terminal fructose**	37.97	42.23	40.96	30.88	43.15	32.14	37.21	37.81
**Terminal glucose**	55.47	51.92	49.08	37.62	51.16	49.94	55.32	29.04
**1-Fructose**	3.52	3.07	6.26	17.61	2.72	11.53	4.30	19.28
**6-Fructose**	0.82	1.08	1.52	6.40	1.32	2.30	1.30	6.00
**6-Glucose**	2.21	1.70	2.19	5.96	1.65	4.09	1.87	5.87
**1,6-Fructose (branched fructose)**	nd	nd	nd	1.53	nd	nd	nd	2.01
**Total (%)**^1^	100.00	100.00	100.00	100.00	100.00	100.00	100.00	100.00

OTT1-OTT4 refers to oligofructans from leaf tips of T1-T4 treated plants. OBT1-OBT4 refers to oligofructans from leaf bases of T1-T4 treated plants. The rest of the signals are not considered since those signals are most-likely contaminants. **nd,** not detected. The data represent the average of three different leaves from three different plants.

^**1**^, corresponds to at least 60% of the total ions obtained by GC-MS

The results from Table 4 show that the T4 tips contain longer oligofructans, as internal sugars such as 1-fructose, 6-fructose, 6-glucose and 1,6-fructose (branched fructose) are increased, whereas both terminal glucose and terminal fructose are decreased compared to T1 tips.

The chromatograms of oligofructans obtained from bases (chromatograms are not shown, Table 4) show similar results to those obtained in tips. Both terminal glucose and terminal fructose decreased in T4 plants compared with T1 plants, whereas the amounts of 1-fructose, 6-fructose, 6-glucose increased with water deficit. Branched fructans (1,6-fructose) were only detected inT4 plants.

The proportions between linear and branched oligofructans are shown in Table 5. Linear oligofructans were estimated by the sum of terminal fructose, terminal glucose, 1-fructose and 6-fructose, whereas branched oligofructans were estimated by the sum of 6-glucose and 1,6 branched fructoses. The results shown were obtained from plants growing at the Las Cardas Experimental Station plus samples taken from plants grown under greenhouse conditions, both of which shared the same glycosidic linkages (data not shown). The linear/branched oligofructan ratio falls with increasing water deficit in the tips and bases, indicating that the relative proportion of branched oligofructans increases when water becomes more limiting.

**Table 5:** Proportions of different glycosidic linkages of oligofructans from leaf tips and bases of Aloe vera plants subjected to different water treatments.

	Water Treatments							
Ratio between glycosidic linkages	OTT1	OTT2	OTT3	OTT4	OBT1	OBT2	OBT3	OBT4
**Linear / Branched**	21.73	22.50	1.80	7.77	23.27	10.86	16.83	4.34
**Terminal Glucose / 6-Glucose**	33.53	17.85	9.66	5.23	42.30	7.29	9.73	3.05
**Total Fructose / Total Glucose**	1.62	1.21	1.82	1.92	1.80	2.03	1.94	2.24

Linear: sum of terminal fructose, terminal glucose, 1-fructose and 6-fructose. Branched: sum of 6-glucose and branched 1,6-fructose. OTT1-OTT4 corresponds to oligofructans from leaf tips and the respective water treatment. OBT1-OBT4 corresponds to oligofructans from the leaf bases and their respective water treatment.

#### Glycosidic linkages of polyfructans

**Fig 10:**
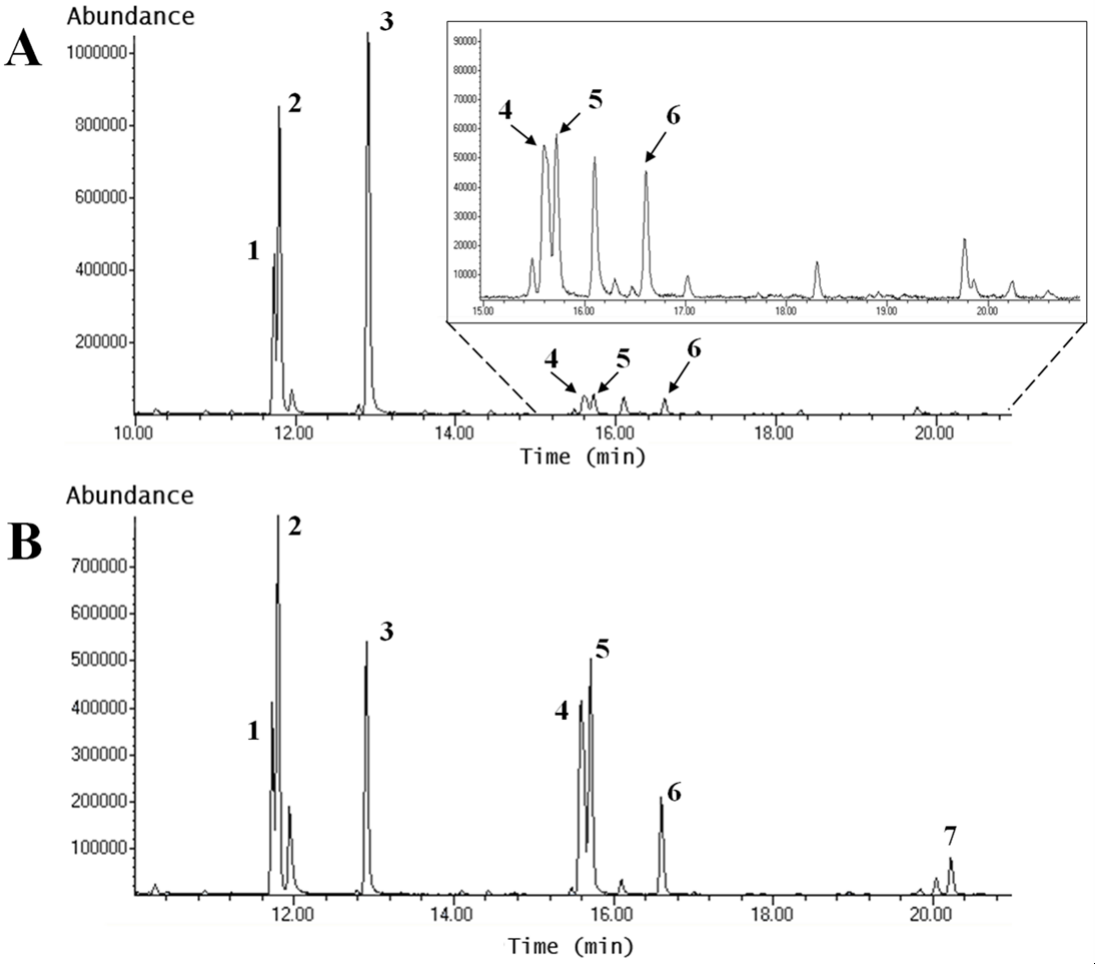
PMAA profiles of polyfructans from Aloe vera leaf tips of plants under different water treatments. The figure shows the results for T1 **(A)** and T4 **(B)** plants. **A)** Chromatogram of the PMAA of oligofructans from leaf tips of T1 plants. **B)** Chromatogram of the PMAA of oligofructans from leaf tips of T4 plants. Peaks **1** and **2:** terminal fructose, **3:** terminal glucose, **4:** 1-fructose and 6-fructose, **5:** 1-fructose, **6:** 6-glucose and **7:** 1,6-fructose (branched fructose). The insert correspond to the amplified chromatogram region shown with dotted lines.

Using the chromatograms obtained (Fig 10A and 10B), the glycosidic bond composition of polyfructans was determined in tips of plants subjected to the four water treatments (Table 6). The PMAA signals of bases were identical to those found for oligofructans present in tips and bases, identifying the same glycosidic linkages between fructose and glucose, with the same retention times (data not shown). As in the case of the oligofructans of Aloe vera, the intensity of 1-fructose, 6-fructose and 6-glucose in tips and bases of T4 plants increases with respect to T1 plants, while 1,6-fructose was only observed in T3 and T4 plants. In contrast, terminal fructose and terminal glucose decreased with water restrictions.

**Table 6:** Glycosidic bond composition (molar %) of the polyfructans of leaf tips and bases of Aloe vera plants subjected to different water treatments.

	Water treatment (molar %)							
Glycosidic linkages	PTT1	PTT2	PTT3	PTT4	PBT1	PBT2	PBT3	PBT4
**Terminal fructose**	4.,83	54.67	4.,00	28,32	50.45	-	34.05	41.28
**Terminal glucose**	4.31	35.97	2.,80	18,06	40.33	-	17.22	29.80
**1-Fructose**	3.55	4.55	17.51	29,28	3.70	-	32.95	17.76
**6-Fructose**	2.30	2.55	4.79	13,02	3.26	-	5.14	4.78
**6-Glucose**	2.00	2.26	4.95	7,95	2.26	-	7.74	5.13
**1,6- Fructose (branched fructose)**	nd	nd	0.95	3,36	nd	-	2.89	1.26
**Total (%)**^1^	100.00	100.00	100.00	100.00	100.00	-	100.00	100.00

PTT1-PTT4 refers to polyfructans from leaf tips of T1-T4 treated plants. PBT1, PBT3 and PBT4 refer to polyfructans from leaf bases of T1, T3 and T4 treated plants. The rest of the signals are not considered since those signals are most-likely contaminants. **nd,** not detected in the sample. GC-MS chromatograms of T2 leaf base samples (PBT2) were not obtained.

^**1**^, corresponds to at least 60% of the total ions obtained by GC-MS.

The proportion between linear and branched polyfructans was estimated (Table 7), as described for oligofructans. As in the case of the oligofructans, the proportion between linear and branched glycosidic linkages, as well as the proportion between terminal glucose and 6-glucose decreases with water deficit in both tips and bases.

**Table 7:** Proportions of different glycosidic linkages of polyfructans from leaf tips and bases of Aloe vera plants subjected to different water treatments.

	Water Treatments						
Ratio between glycosidic linkages	PTT1	PTT2	PTT3	PTT4	PBT1	PBT3	PBT4
**Linear / Branched**	49.02	43.26	15.96	7.84	43.33	8.41	14.66
**Terminal Glucose / 6-Glucose**	22.16	15.92	5.21	2.27	17.88	2.22	5.81
**Total Fructose / Total Glucose**	1.16	1.62	2.25	2.84	1.35	3.01	1.86

Linear: sum of terminal fructose, terminal glucose, 1-fructose and 6-fructose. Branched: sum of 6-glucose and branched 1,6-fructose. PTT1-PTT4 corresponds to polyfructans from leaf tips and the respective water treatment. PBT1, PBT3 and PBT4 refer to polyfructans from leaf bases of T1, T3 and T4 treated plants. GC-MS chromatograms of T2 leaf base samples (PBT2) were not obtained.

## Discussion

As a CAM plant, Aloe vera synthesizes sugars efficiently. Here, we show that under water deficit conditions, sugar levels increase in leaves of this species. This rise could be due to increased synthesis, reduced mobilization or a combination of both processes. Like other CAM species, Aloe vera assimilates CO_2_ very efficiently, with almost no photorespiration [2]. As a consequence, sugars and thus polysaccharides are efficiently synthesized, serving as molecules to store water during the drought periods of the arid regions [1, 2, 3]. Therefore, in drought, sugar synthesis may indeed occur in Aloe vera and may explain how the leaves maintain such a succulent appearance [8]. Experiments undertaken in our laboratory show that the *1-sst* gene encoding the sucrose: sucrose 1-fructosyl transferase and the *6G-fft* gene, encoding the 6-glucose-fructan-fructosyl transferase, both increase in expression in Aloe vera plants subjected to water stress (data not shown). Under water restrictions, sugars function as osmolytes, performing a role in osmotic adjustment (OA) [8, 4]. OA is the decrease of the cell water potential due to a net accumulation of solutes or osmolytes, as a response to water deficit. The accumulation of osmolytes occurs in order to maintain a water gradient favorable towards the interior of the cell [48]. Therefore, with an efficient OA, the plant is able to tolerate drought conditions better.

On the other hand, not all macromolecules increase in amounts in Aloe vera plants subjected to water stress. Indeed, in the same gram of dry weight of leaf tips and bases, while the sugars increase, protein levels fall. This is expected because a portion of the housekeeping proteins are degraded due to the role of ubiquitin in removing proteins denatured by stress [49].

Some of the osmolytes accumulated in Aloe vera are fructans. In a previous study [4], we reported that proline, soluble sugars, total sugars, and fructans increase in Aloe vera plants under water restrictions. The quantification results obtained in the present study with the modified colorimetric anthrone test [50] confirm our previous findings [4]. Oligofructans and polyfructans increase gradually with water stress, in both tips and bases. However, the accumulation of these polysaccharides in plants with normal irrigation (T1) is greater in leaf bases than in tips, even though photosynthesis occurs in Aloe vera mainly in the upper portion of the leaf. This is not surprising because the classical role of fructans is considered to be as storage molecules, and, therefore, functioning as a carbon source for the plant. Being a monocot plant, Aloe vera stores fructans in leaves, mainly in the base, whereas dicots do so in underground organs [51].

Under water restriction conditions, as experienced by T2, T3, and T4 plants, oligofructans and polyfructans rise gradually with water deficit. However, in T4 plants, oligo- and polyfructans increase more in tips than in bases. Specifically, oligofructans are 2.35-fold and 1.70-fold more abundant in T4 versus T1 plants, in leaf tips and bases, respectively, whilst for polyfructans, the corresponding values are 5.85-fold more in tips, compared to 2.4-fold more in the bases, comparing T4- and T1-irrigated plants.

The estimation of oligo- and polyfructan quantities performed by TLC analyses also coincides with the data obtained with the modified colorimetric method of anthrone [50]. The greater increase of oligofructans in tips probably occurs because the tip is the part of the leaf with a greater density of chloroplasts and is thus more photosynthetically active. Instead, the base appears to serve as the storage part of the leaf. Therefore, tips require more protection against water stress, and thus higher amounts of fructans. Both oligo- and polyfructans are water soluble and excellent osmolytes and, as a consequence, the substantial increase in these compounds in tips could protect photosynthetic functions [52, 53].

The TLC analyses show that the intensity of the spots of oligo- and polyfructans increase with water restriction in all DPs. This analysis also shows that in all samples there are two visible trisaccharides, 1-kestose, which gives rise to the inulin-type of fructans, and neo-kestose which is the trisaccharide present in neo-levans and neo-fructans. The results of our analyses demonstrate that both trisaccharides increase with water deficit. In the TLC plates, neo-kestose increases with water stress more than 1-kestose. 6-Kestose, the trisaccharide of the levan-type of fructans and graminans [24, 54, 55] was not detected. The presence of 1-kestose and neo-kestose confirms that Aloe vera most probably synthesizes inulin, neo-inulin and neo-levan types of fructans [56], whilst the appearance of 1,6-fructose confirms that under severe water stress, Aloe vera also manufactures neo-fructans to a detectable level. This metabolic evidence is in line with that of other species of the order Asparagales, such as onion, garlic, asparagus and agave, which also synthesize these four types of fructans [21, 23, 25, 24]. Because neo-kestose increases more than 1-kestose during water stress, neo-levans and neo-fructans are probably synthesized preferentially by Aloe vera under these conditions. If so, the enzyme transferring a fructofuranosyl radical to C6 of the glucose residue of sucrose (6G-FFT), proper of the neo-levans and neo-fructans, most likely increases in activity and/or amount under water stress conditions, a theory which we are currently testing.

The TLC analyses also reveal that the DP of oligo- and polyfructans rises with increasing water deficit. In oligo- and polyfructans, the increase in DP occurs along with a decrease in free sucrose in both parts of the leaves. Probably, the sucrose is being used as a donor sugar for the synthesis of the trisaccharide 1-kestose by 1-SST and later as a sugar acceptor in conjunction with 1-kestose to produce neo-kestose by 6G-FFT, as reported by Cortés-Romero *et al*. [57]. In Agave the same authors reported that 6G-FFT uses directly sucrose to produce *in vitro* the fructan neo-kestose [57].

The increase in DP initially observed by the TLC analyses was corroborated by MALDI-ToF-MS analyses, indicating that there is probably a structural modification with mass increment of the fructans from plants subjected to water deficit. Both the increment in amount and in the DP of fructans could play an important role in the protection of the leaf cells during water stress.

A more accurate quantification of fructans was performed by derivatization into alditol acetates of the sugar residue components of the Aloe vera fructans, followed by GC-MS analyses. As a product of the keto-enolic tautomerism of fructose, we detected glucose and mannose as sorbitol and mannitol, respectively, in the GC-MS analysis [47, 58]. The sum of these two sugar residues was used to calculate the total amount of oligo- and polyfructans. Similar to the results obtained with the modified colorimetric anthrone test, we found that oligo- and polyfructans increased with increasing water deficit in the plants. Overall, bases have more fructans than tips, but the upper part of the leaf probably accumulates proportionally more oligofructans and polyfructans under water restriction conditions (T2, T3, and T4) compared to the bases.

To confirm that oligo- and polyfructans of the neo-levan and neo-fructans structures are present and increase during water stress in Aloe vera plants, analyses were performed by PMAA derivatization of sugar residues to determine the glycosidic linkages by GC-MS. The data obtained (Tables 4, 5, 6 and 7), indicate that the glycosidic bonds are those commonly present in plant fructans. The glycosidic bonds found were of the types 1-fructose [β-(2→1)], 6-fructose [β-(2→6)], 6-glucose [fructose-β-(2→6)-glucose] and 1,6-fructose [β-(2→1)+β-(2→6)], the latter corresponding to branched fructose [25, 59, 60, 61]. The data also show that the fructans of plants propagated under water restriction suffer an increase in 1-fructose, 6-fructose, and 6-glucose glycosidic bonds, while the terminal glucose and terminal fructose decrease. 1-Fructose, a linkage found in the linear type of fructans, increases up to 5 times in T4 plants compared with T1 plants in the tip portion of the leaf in oligofructans, and 8.25 times in polyfructans. This result indicates that inulin and neo-inulin increase in amount during water deficit. 6-Fructose, which is also found in the linear part of fructans, increases 7.0 times in oligofructans in the tip portion of T4 plants, and 5.66 times in the case of the polyfructans in the same portion of T4 leaves. These results indicate that neo-levans and neo-fructans are present under water stress conditions. Therefore, since 1-fructose and 6-fructose increase under water restrictions, we conclude that both oligo- and polyfructans, are increasing in DP with water deficit. 6-Glucose, a linkage found in neo-levans, neo-inulins and neo-fructans, also augments with water deficit. Therefore, these results indicate that these three types of fructans are present and probably increase in quantities with water restrictions (by 2.5 times in tips and 3.4 times in bases in the case of polyfructans of T3 plants).

However, 1,6-fructose (a branched fructan) appears only in plants under the most severe water restrictions, i.e. in T3 and T4 plants. This is a linkage that forms a branch point in the fructose chain, typical of the neo-fructans [62, 63]. Therefore, it appears that the neo-fructans are synthesized in Aloe vera plants subjected to a severe drought condition. Because 6-kestose was never found in the TLC analyses, we believe that levans and graminans are not present in Aloe vera, even though 1,6-fructose is a glycosidic linkage typical of the neo-fructans and also present in the graminan structure. However, in the graminans there is no branched glucose at the C6 linkage of the glucose [64]. 1-Fructose and 6-fructose are linkages of the linear type of fructans (inulin, neo-inulin and neo-levans), and we conclude that these predominate in Aloe vera plants under normal irrigation (T1) and mild water deficit (T2 and T3). We also ascertain that the branched neo-fructan structures appear in oligofructans of T4 plants and in polyfructans of T3 and T4 plants.

The PMAA analyses corroborate the increase in DP of fructans in plants under water stress, because terminal residues decreased under these conditions, increasing the amounts of 1-fructose and 6-fructose. A correlation between fructan DP and the membrane protection function of fructans has been reported [18, 65]. Fructans with a higher DP protect membranes better against desiccation than fructans with a lower DP. Additionally, large DP fructans are better cryoprotectants for cells and proteins [66]. However, mixtures of fructans of high DP with fructans of lower DP seem to be more effective in membrane protection than fructans of larger DP alone [18, 65]. Recently it has been reported that fructans of low DP move through the phloem, acting as signaling compounds in plants under stress [15]. Given the important role that fructans have as protective molecules during stress, efforts to determine the precise DP of Aloe vera fructans in water deficit are currently underway.

Fructan accumulation during cold [11, 67], salt and drought stress [16, 20] has been well-established. Evidence that fructans protect plants from freezing and drought comes from the fact that these polysaccharides are abundant in plants of cold and arid climates, whilst plants of tropical environments do not accumulate these polysaccharides [15, 20]. The physiological role of protection of fructans against cold and drought has been reinforced by the demonstration that transgenic plants transformed with the fructan transferase genes are able to synthesize fructans, and are more tolerant to these abiotic stress conditions [67, 68, 69]. Fructans, due to the repetitive hydroxyl groups of their molecules, are capable of hydrogen bonding with the polar regions of the membrane phospholipids, and with the hydrophilic amino acids of proteins [24, 70, 71]. In other words, the sugars and the fructans replace water molecules during water loss. Fructans also act as antioxidant molecules during oxidative stress, a consequence of cold and drought, by scavenging ·OH radicals [72, 73]. These harmful reactive oxygen species form in the vacuolar compartment [72], where fructans also accumulate [15].

In summary,

1. Aloe vera plants under water deficit synthesize more fructans and of a higher DP.
2. Glycosidic linkage determination of these polymers shows that probably in plants of water treatments T1, T2 and T3 there is a mix of inulin, neo-inulin and neo-levans, and these increase considerably with increasing water deficit.
3. T3 and T4 plants synthesize oligo and polyfructans with a neo-fructan structure.

There are several reports of fructan synthesis in plants subjected to water deficit. In *Cichorium intybus* (chicory) seedlings, *1-sst* expression increases under drought [16] and in *Lolium perenne* hormones like abscisic acid increase the activity of enzymes responsible for fructan synthesis (1-SST and 6G-FFT), although without changes in fructan levels [74]. In *Agave tequilana* and *Agave inaequidens*, under biotic and abiotic stresses and with salicylic acid application, the expression of genes encoding for fructosyltransferases (1-SST and 1-FFT) increase [75]. Finally, and of particular relevance, the transition of the amphibious freshwater plant *Littorella uniflora* from an aquatic to a terrestrial environment is accompanied by increased fructan levels [76].

Alterations in fructan structure in terms of DP induced by drought and chilling conditions have also been reported. In wheat, roots accumulate fructans of higher DP under chilling conditions compared to leaves where fructans accumulate with a lower DP [77]. During wheat kernel development from anthesis until maturity, a loss of water takes place during grain filling. At this stage, the fructan DP decreases due to an increase in the activity of fructan hydrolase enzymes [78]. In the Cerrado species *Chrysolaena obovata*, water stress induces the presence of inulin of smaller DP, while re-watering the plant leads to the accumulation of inulin of larger DP [79]. Additionally, as *A*. *tequilana* plants, fructans become longer and more branched with the age of the plant [80]. However, to our knowledge, the synthesis and the protective role of neo-fructans under extreme water deficit has not been previously reported.

Why does Aloe vera synthesize neo-fructans under severe water stress? Probably the branched structure of these molecules has more interactions by hydrogen bonds with the hydrophilic phospholipid heads and with hydrophilic amino acids, protecting the cellular structure better under desiccation conditions [24, 70, 71]. In addition, the molecular control of these structural changes has yet to be addressed, and we speculate that different fructan transferases are responsible, at least in part, for the modifications observed.

## References

[1] Cushman JC. 2001 Crassulacean Acid Metabolism. A Plastic Photosynthetic Adaptation to Arid Environments. Plant Physiol. 127: 1439–1448. 11743087 PMC1540176

[2] Cushman JC, Borland AM. 2002 Induction of Crassulacean acid metabolism by water limitation. Plant Cell and Environ. 25: 295–310. 10.1046/j.0016-8025.2001.00760.x 11841671

[3] Silva H, Sagardia S, Seguel O, Torres C, Tapia C, Franck N, et al 2010 Effect of water availability on growth and water use efficiency for biomass and gel production in Aloe Vera (Aloe barbadensis M.). Ind Crop Prod. 31: 20–27.

[4] Delatorre-Herrera J, Delfino I, Salinas C, Silva H, Cardemil L. 2010 Irrigation restriction effects on water use efficiency and osmotic adjustment in Aloe vera plants (*Aloe barbadensis* Miller). Agr Water Manage. 97: 1564–1570.

[5] Vinocur B, Altman A. 2005 Recent advances in engineering plant tolerance to abiotic stress: achievements and limitations. Curr Opin Biotech. 16: 123–132. 15831376 10.1016/j.copbio.2005.02.001

[6] Seki M, Umezawa T, Urano K, Shinozaki K. 2007 Regulatory metabolic networks in drought stress responses. Curr Opin Plant Biol. 10: 296–302. 17468040 10.1016/j.pbi.2007.04.014

[7] Rekarte-Cowie I, Ebshish OS, Mohamed KS, Pearce RS. 2008 Sucrose helps regulate cold acclimation of *Arabidopsis thaliana*. J Exp Bot. 59: 4205–4217. 10.1093/jxb/ern262 18980951 PMC2639016

[8] Ogburn MR, Edwards EJ. 2010 The Ecological Water-Use Strategies of Succulent Plants. Adv Bot Res. 55: 179–225.

[9] Jeong BR, Housley TL 1990 Fructan Metabolism in Wheat in Alternating Warm and Cold Temperatures. Plant Physiol. 93: 902–906. 16667599 10.1104/pp.93.3.902 PMC1062607

[10] Tognetti JA, Salerno GL, Crespi MD, Pontis HG. 1990 Sucrose and fructan metabolism of different wheat cultivars at chilling temperatures. Physiol Plantarum. 78: 554–559.

[11] Livingston DP III, Henson CA. 1998 Apoplastic Sugars, Fructans, Fructan Exohydrolase, and Invertase in Winter Oat: Responses to Second-Phase Cold Hardening. Plant Physiol. 116: 403–408.

[12] del Viso F, Puebla AF, Fusari CM, Casabuono AC, Couto AS, Pontis HG, et al 2009 Molecular Characterization of a Putative Sucrose:Fructan 6-Fructosyltransferase (6-SFT) of the Cold-Resistant Patagonian Grass *Bromus pictus* Associated With Fructan Accumulation Under Low Temperatures. Plant Cell Physiol. 50: 489–503. 10.1093/pcp/pcp008 19153157

[13] Koster KL, Webb MS, Bryant G, Lynch DV. 1994 Interactions between soluble sugars and POPC (1-palmitoyl-2-oleoylphosphatidylcholine) during dehydration: vitrification of sugars alters the phase behavior of the phospholipid. Biochim Biophys Acta. 1193: 143–150. 8038184 10.1016/0005-2736(94)90343-3

[14] Tsvetkova NM, Phillips BL, Cowe LM, Crowe JH, Risbud SH. 1998 Effect of Sugars on head group Mobility in Freeze-Dried Dipalmitoylphosphatidylcholine Bilayers: Solid-State 31P NMR and FTIR Studies. Biophys J. 75: 2947–2955. 9826615 10.1016/S0006-3495(98)77736-7 PMC1299966

[15] Van den Ende W. 2013 Multifunctional fructans and raffinose family oligosaccharides. Front Plant Sci. 4: 247, 10.3389/fpls.2013.00247 23882273 PMC3713406

[16] De Roover J, Vandenbranden K, Van Laere A, Van den Ende W. 2000 Drought induces fructan synthesis and 1-SST (sucrose: sucrose fructosyltransferase) in roots and leaves of chicory seedlings (*Cichorium intybus* L.). Planta. 210: 808–814. 10805453 10.1007/s004250050683

[17] Amiard V, Morvan-Bertrand A, Billard JP, Huault C, Keller F, Prud’homme MP. 2003 Fructans, But Not the Sucrosyl-Galactosides, Raffinose and Loliose, Are Affected by Drought Stress in Perennial Ryegrass. Plant Physiol. 132: 2218–2229. 12913176 10.1104/pp.103.022335 PMC181305

[18] Vereyken IJ, van Kuik JA, Evers Rijken PJ, de Kruijff B. 2003 Structural Requirements of the Fructan-Lipid Interaction. Biophys J. 84: 3147–3154. 12719244 10.1016/s0006-3495(03)70039-3 PMC1302875

[19] Valluru R, Van den Ende W. 2008 Plant fructans in stress environments: emerging concepts and future prospects. J Exp Bot. 59: 2905–2916. 10.1093/jxb/ern164 18603617

[20] Hendry G. 1993 Evolutionary origins and natural functions of fructans—a climatological, biogeographic and mechanistic appraisal. New Phytol. 123: 3–14.

[21] Vijn I, Smeekens S. 1999 Fructan: More Than a Reserve Carbohydrate? Plant Physiol. 120: 351–359. 10364386 10.1104/pp.120.2.351 PMC1539216

[22] Bieleski R. 1993 Fructan Hydrolysis Drives Petal Expansion in the Ephemeral Daylily Flower. Plant Physiol. 103: 213–219. 12231928 10.1104/pp.103.1.213 PMC158965

[23] Van den Ende W, De Coninck B, Van Laere A. 2004 Plant fructan exohydrolases: a role in signaling and defense? Trends Plant Sci. 9: 523–528. 15501176 10.1016/j.tplants.2004.09.008

[24] Livingston DP III, Hincha DK, Heyer AG. 2009 Fructan and its relationship to abiotic stress tolerance in plants. Cell Mol Life Sci. 66: 2007–2023. 10.1007/s00018-009-0002-x 19290476 PMC2705711

[25] Mancilla-Margalli NA, López MG. 2006 Water-Soluble Carbohydrates and Fructan Structure Patterns from *Agave* and *Dasylirion* Species. J Agr Food Chem. 54: 7832–7839. 17002459 10.1021/jf060354v

[26] Ni Y, Turner D, Yates KM, Tizard I. 2004 Isolation and characterization of components of Aloe vera L. leaf pulp. Int Immunopharmacol. 4: 1745–1755. 15531291 10.1016/j.intimp.2004.07.006

[27] Chase MW. 2004 Monocot Relationship: An overview. Am J Bot. 91: 1645–1655. 10.3732/ajb.91.10.1645 21652314

[28] Chase MW, Reveal JL, Fay MF. 2009 A subfamilial classification for the expanded asparagalean families Amaryllidaceae, Asparagaceae and Xanthorrhoeaceae. Bot J Linn Soc. 161: 132–136.

[29] Gonzalez G, Salinas C, Allende M, Cardemil L. 2014. In vivo evaluation of immune-modulatory and regenerative properties of polysaccharides from Aloe barbadensis Miller. Segundo Congreso Latinoamericano de Plantas Medicinales, August 12–14. Santiago, Chile.

[30] Roberfroid M. 2007 Prebiotics: The Concept Revisited. J Nutr. 137: 830S–837. 17311983 10.1093/jn/137.3.830S

[31] Quezada MP, Salinas C, Gotteland M, Cardemil L. 2014. Aloe barbadensis Miller, a novel source for prebiotic polysaccharides. Segundo Congreso Latinoamericano de Plantas Medicinales, August 12–14. Santiago, Chile.

[32] Cardemil L, Reinero A. 1982 Changes of *Araucaria araucana* reserves during germination and early seedling growth. Can J Botany. 60: 1629–1638.

[33] Cairns AJ, Pollock CJ. 1988 Fructan biosynthesis in excised leaves of *Lolium temulentum* L. I. Chromatographic characterization of oligofructans and their labelling patterns following ^14^CO_2_ feeding. New Phytol. 109: 399–405.

[34] Dische R. 1962 Color reactions of carbohydrates *In* Whistler y R.L. Wolfrom M.L. (eds.), Methods in Carbohydrate Chemistry. pp 477–512. Academic Press, New York, USA.

[35] Jermyn MA. 1956 A new method for determining Ketohexoses in the presence of Aldohexoses. Nature. 177: 38–39.

[36] Van Handel E. 1967 Determination of fructose and fructose-yielding carbohydrates with cold anthrone. Anal Biochem. 19: 193–194. 6048683 10.1016/0003-2697(67)90152-2

[37] Machado de Carvalho MA, Dietrich SMC. 1993 Variation in fructan content in the underground organs of *Vernonia herbacea* (Vell.) Rusby at different phenological phases. New Phytol. 123: 735–740.

[38] Bradford MM. 1976 A rapid and sensitive method for the quantitation of microgram quantities of protein utilizing the principle of protein-dye binding. Anal Biochem. 72:248–254. 942051 10.1016/0003-2697(76)90527-3

[39] Spollen WG, Nelson CJ. 1988 Characterization of Fructan from Mature Leaf Blades and Elongation Zones of Developing Leaf Blades of Wheat, Tall Fescue, and Timothy. Plant Physiol. 88: 1349–1353. 16666465 10.1104/pp.88.4.1349 PMC1055763

[40] Wise CS, Dimler RJ, Davis HA, Rist CE. 1955 Determination of Easily Hydrolyzable Fructose Units in Dextran Preparations. Anal Chem. 27: 33–35.

[41] Maslen SL, Goubet F, Adam A, Dupree P, Stephens E. 2007 Structure elucidation of arabinoxylan isomers by normal phase HPLC–MALDI-TOF/TOF-MS/MS. Carbohyd Res. 342: 724–735. 10.1016/j.carres.2006.12.007 17208205

[42] Cardemil L, Wolk CP. 1976 The polysaccharides from heterocyst and spore envelopes of a blue-green alga. Methylation analysis and structure of the backbones. J Biol Chem. 251: 2967–2975. 818084

[43] York W, Darvill AG, McNeil M, Albersheim P. 1985 3-deoxy-D-*manno*-2-octulosonic acid (KDO) is a component of rhamnogalacturonan II, a pectic polysaccharide in the primary cell wall of plants. Carbohyd Res. 138: 109–126.

[44] Ciucanu I, Kerek F. 1984 A simple and rapid method for the permethylation of Carbohydrate. Carbohyd Res. 131: 209–217.

[45] Ciucanu I. 2006 Per-*O*-methylation reaction for structural analysis of carbohydrates by mass spectrometry. Anal Chim Acta. 576: 147–155. 17723627 10.1016/j.aca.2006.06.009

[46] Foster C E, Martin T, Pauly M. 2010. Comprehensive compositional analysis of Plant Cell Walls (lignocellulosic biomass); Part II: carbohydrates. J Vis Exp. 37 10.3791/1837 PMC3145335 20228730

[47] Carpita NC, Shea EM. 1989 Linkage structure of carbohydrates by gas chromatography-mass spectrometry (GC-MS) of partially methylated alditol acetates Biermann y En C.J. McGinnis G.D. (eds.), Analysis of Carbohydrates by GLC and MS. pp 157–216. CRC Press, Boca Raton, Florida, USA.

[48] Morgan JM. 1984 Osmoregulation and water stress in higher plants. Annu Rev Plant Physiol. 35: 299–319.

[49] Huerta C, Freire M, Cardemil L. 2013 Expression of hsp70, hsp100 and ubiquitin in Aloe barbadensis Miller under direct heat stress and under temperature acclimation conditions. Plant Cell Rep. 32: 293–307, 10.1007/s00299-012-1363-4 23111788

[50] Jermyn MA. 1975 Increasing the Sensitivity of the Anthrone Method for Carbohydrate. Anal Biochem. 68: 332–335. 1190448 10.1016/0003-2697(75)90713-7

[51] Slewinski TL. 2012 Nonstructural carbohydrate partitioning in grass stems: a target to increase yield stability, stress tolerance, and biofuel production. J Exp Bot. 63: 4647–4670. 10.1093/jxb/ers124 22732107

[52] Zhang J, Nguyen H, Blum A. 1999 Mechanisms of plant desiccation tolerance. J Exp Bot. 50: 291–302.

[53] Burg MB, Ferraris JD. 2008 Intracellular Organic Osmolytes: Function and Regulation. J Biol Chem. 283: 7309–7313. 10.1074/jbc.R700042200 18256030 PMC2276334

[54] Altenbach D, RudinoPinera E, Olvera C, Boller T, Wiemken A, Ritsema T. 2009 An acceptor substrate binding site determining glycosyl transfer emerges from mutant analysis of a plant vacuolar invertase and a fructosyltransferase. Plant Mol Biol. 69: 47–56. 10.1007/s11103-008-9404-7 18821058 PMC2709226

[55] Van den Ende W, Coopman M, Clerens S, Vergauwen R, Le Roy K, Lammens W, et al 2011 Unexpected presence of graminan and levan type fructans in the evergreen frosthardy eudicot Pachysandra terminalis (Buxaceae). Purification, cloning and functional analysis of a 6SST/6SFT enzyme. Plant Physiol. 155: 603–614. 10.1104/pp.110.162222 21037113 PMC3075768

[56] Ritsema T, Smeekens S. 2003 Fructans: beneficial for plants and humans. Curr Opin Plant Biol. 6: 223–230. 12753971 10.1016/s1369-5266(03)00034-7

[57] Cortés-Romero C, Martínez-Hernández A, Mellado-Mojica E, López MG, Simpson J. 2012 Molecular and Functional Characterization of Novel Fructosyltransferases and Invertases from *Agave tequilana*. PLoS One. 7: e35878 10.1371/journal.pone.0035878 22558253 PMC3340406

[58] Abdel-Akherj M, Hamilton K, Smith F. 1951 The Reduction of Sugars with Sodium Borohydride. J Am Chem Soc. 73: 4691–4692.

[59] López MG, Mancilla-Margalli NA, Mendoza-Díaz G. 2003 Molecular Structures of Fructans from *Agave tequilana* Weber var. *azul*. J Agr Food Chem. 51: 7835–7840. 14690361 10.1021/jf030383v

[60] Wack M, Blaschek W. 2006 Determination of the structure and degree of polymerisation of fructans from *Echinacea purpurea* roots. Carbohyd Res. 341: 1147–1153. 10.1016/j.carres.2006.03.034 16631147

[61] Ravenscroft N, Cescutti P, Hearshaw MA, Ramsout R, Rizzo R, Timme EM. 2009 Structural Analysis of Fructans from Agave americana Grown in South Africa for Spirit Production. J Agr Food Chem. 57: 3995–4003. 19348427 10.1021/jf8039389

[62] Sims IM. 2003 Structural diversity of fructans from members of the order Asparagales in New Zealand. Phytochemistry. 63: 351–359. 12737984 10.1016/s0031-9422(03)00132-8

[63] Lammens W, Le Roy K, Schroeven L, Van Laere A, Rabijns A, Van den Ende W. 2009 Structural insights into glycoside hydrolase family 32 and 68 enzymes: functional implications. J Exp Bot. 60: 727–740. 10.1093/jxb/ern333 19129163

[64] Yoshida M, Kawakami A, Van den Ende W. 2007 Graminan metabolism in cereals: wheat as a model system In Recent Advances in Fructooligosaccharides Research. Edited by Shiomi N, Benkeblia N, Onodera S. pp. 201–212. Research Signpost, Kerala, India.

[65] Hincha DK, Livingston DP III, Premakumar R, Zuther E, Obel N, Cacela C, et al 2007 Fructans from oat and rye: Composition and effects on membrane stability during drying. Biochim Biophys Acta. 1768: 1611–1619. 17462587 10.1016/j.bbamem.2007.03.011

[66] Rodríguez Furlán LT, Herrera PA, Pérez Padilla A, Ortiz Basurto R I, Campderrós ME. 2014 Assessment of agave fructans as lyoprotectants of bovine plasma proteins concentrated by ultrafiltration. Food Res Int. 56: 146–158.

[67] Li HJ, Yang AF, Zhang XC, Gao F, Zhang JR. 2007 Improving freezing tolerance of transgenic tobacco expressing sucrose:sucrose 1-fructosyltransferase gene from *Lactuca sativa*. Plant Cell Tiss Org. 89: 37–48.

[68] Kawakami A, Sato Y, Yoshida M. 2008 Genetic engineering of rice capable of synthesizing fructans and enhancing chilling tolerance. J Exp Bot. 59: 793–802. 10.1093/jxb/erm367 18319240

[69] Bie X, Wang K, She M, Du L, Zhang S, Li J. et al (2012) Combinational transformation of three wheat genes encoding fructan biosynthesis enzymes confers increased fructan content and tolerance to abiotic stresses in tobacco. Plant Cell Rep. 31: 2229–2238 10.1007/s00299-012-1332-y 22911265

[70] Hoekstra FA, Golovina EA, Buitink J. 2001 Mechanisms of plant desiccation tolerance. Trends Plant Sci. 6: 431–438. 11544133 10.1016/s1360-1385(01)02052-0

[71] Vereyken IJ, Chupin V, Demel RA, Smeekens SCM, de Kruijff B. 2001 Fructans insert between the headgroups of phospholipids. Biochim Biophys Acta. 1510: 307–320. 11342168 10.1016/s0005-2736(00)00363-1

[72] Peshev D, Vergauwen R, Moglia A, Hideg E, Van den Ende W. 2013 0p J Exp Bot. 64: 1025–1038. 10.1093/jxb/ers377 23349141 PMC3580814

[73] Stoyanova S, Geuns J, Hideg E, Van den Ende W. 2011 The food additives inulin and stevioside counteract oxidative stress. Int J Food Sci Nutr. 62: 207–214. 10.3109/09637486.2010.523416 21043580

[74] Gasperl A, Morvan-Bertrand A, Prud’homme MP, van der Graaff E, Roitsch T. 2016 Exogenous Classic Phytohormones Have Limited Regulatory Effects on Fructan and Primary Carbohydrate Metabolism in Perennial Ryegrass (Lolium perenne L.). Front Plant Sci. 6: 1–19. 10.3389/fpls.2015.01251 PMC4719101 26834764

[75] Suárez-González EM, López MG, Délano-Frier JP, Gómez-Leyva JF. 2014 Expression of the 1-SST and 1-FFT genes and consequent fructan accumulation in *Agave tequilana* and *A*. *inaequidens* is differentially induced by diverse (a)biotic-stress related elicitors. Front Plant Sci. 6: 721 10.3389/fpls.2015.00721 23988562

[76] Robe WE, Griffiths H. 2000 Physiological and photosynthetic plasticity in the amphibious, freshwater plant Littorella uniflora,during the transition from aquatic to dry terrestrial environments. Plant Cell Environ. 23: 1041–1054.

[77] Santoiani CS, Tognetti JA, Pontis HG, Salerno GL. 1993 Sucrose and fructan metabolism in wheat roots at chilling temperatures. Physiol Plantarum. 87: 84–88.

[78] Verspreet J, Hansen AH, Dornez E, Delcour JA, Van den Ende W, Harrison SJ, et al 2015 LC-MS analysis reveals the presence of graminan- and neo-type fructans in wheat grains. J Cereal Sci. 61: 133–138.

[79] Garcia PMA, Hayashi AH, Silva EA, Figueiredo-Ribeiro RdeCL, Carvalho MAM. 2015 Structural and metabolic changes in rhizophores of the Cerrado species *Chrysolaena obovata* (Less.) Dematt. as influenced by drought and re-watering. Front Plant Sci. 6: 721 10.3389/fpls.2015.00721 26442035 PMC4585265

[80] Arrizon J, Morel S, Gschaedler A, Monsan P. 2010 Comparison of the water-soluble carbohydrate composition and Fructan structures of *Agave tequilana* plants of different ages. Food Chem. 122: 123–130.

